# Submicron‐Sized In Situ Osmotic Pressure Sensors for In Vitro Applications in Biology

**DOI:** 10.1002/adhm.202202373

**Published:** 2023-01-13

**Authors:** Wenbo Zhang, Luca Bertinetti, Efe Cuma Yavuzsoy, Changyou Gao, Emanuel Schneck, Peter Fratzl

**Affiliations:** ^1^ Department of Biomaterials Max Planck Institute of Colloids and Interfaces 14476 Potsdam Germany; ^2^ B CUBE Center for Molecular Bioengineering Technical University of Darmstadt 01307 Dresden Germany; ^3^ MOE Key Laboratory of Macromolecular Synthesis and Functionalization Department of Polymer Science and Engineering Zhejiang University Hangzhou 310027 China; ^4^ Department of Physics Technical University of Darmstadt 64289 Darmstadt Germany

**Keywords:** biosensing, imaging, liposomes, resonance energy transfer, semi‐permeable membranes

## Abstract

Physical forces are important cues in determining the development and the normal function of biological tissues. While forces generated by molecular motors have been widely studied, forces resulting from osmotic gradients have been less considered in this context. A possible reason is the lack of direct in situ measurement methods that can be applied to cell and organ culture systems. Herein, novel kinds of resonance energy transfer (FRET)‐based liposomal sensors are developed, so that their sensing range and sensitivity can be adjusted to satisfy physiological osmotic conditions. Several types of sensors are prepared, either based on polyethylene glycol‐ (PEG)ylated liposomes with steric stabilization and stealth property or on crosslinked liposomes capable of enduring relatively harsh environments for liposomes (e.g., in the presence of biosurfactants). The sensors are demonstrated to be effective in the measurement of osmotic pressures in pre‐osteoblastic in vitro cell culture systems by means of FRET microscopy. This development paves the way toward the in situ sensing of osmotic pressures in biological culture systems.

## Graphical abstract

Novel liposome‐based local sensors are developed for in situ measurements of osmotic pressures in cell cultures exploiting the resonance energy transfer (FRET) principle, which has not been achieved so far. With the help of fluorescence microscopy, the feasibility of in situ osmotic pressure imaging in pre‐osteoblastic in vitro cell culture systems is demonstrated.

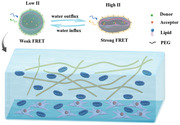

## 1 Introduction

Osmotic pressure is of vital importance in biology, as it can generate forces and modulate the functions of biomolecular assemblies.^[^ ^1^ ^]^ In biological tissues, osmotic pressure is constantly regulated by a balance of hydration and solute concentrations.^[^ ^2^ ^]^ In animals, osmotic pressure in soft connective tissues, articular cartilage, and intervertebral discs is used for mechanical purposes. The osmotic pressure generated by negatively charged proteoglycans and their counterions (e.g., Na^+^, Ca^2+^) contributes to the compressive resistance of the tissues, making it possible to bear loads of several times the body weight.^[^ ^1^, ^3^ ^]^ In the extracellular matrix (ECM), osmotic pressures were reported to be responsible for the conformational changes of molecules such as collagen,^[^ ^1^, ^4^ ^]^ leading to contractile stress in this molecule, and is probably involved in the mineralization process of collagen.^[^ ^5^ ^]^ In a recent work, ECM‐derived osmotic pressure was identified as the driving force for tissue morphogenesis in a epithelium model, the semicircular canal development system.^[^ ^6^ ^]^ At the cell level, changes in the extracellular osmotic environment alter the volume, and hence the physiochemical properties of cells such as cell stiffness, intracellular material concentration, and molecular crowding.^[^ ^7^ ^]^ In turn, the structure/function of the cell nucleus, the gene expression and metabolic activity may be impacted.^[^ ^7b^ ^]^ In some seminal studies, the osmoregulatory responses of the cells were found to induce mesenchymal stem cell differentiation ^[^ ^7a^ ^]^ and growth arrest/reactivation of human metastatic cells.^[^ ^8^ ^]^

Despite the great biological importance of osmotic pressures, little is still known about the distribution and temporal evolution of osmotic pressure in tissues. Conventional methods for osmotic pressure determination rely either on direct measurements with semipermeable membrane systems or on colligative properties (e.g., freezing point/ vapor pressure lowering) and are therefore inapplicable to spatially resolved in situ or in vivo measurements.^[^ ^9^ ^]^ Instead, osmotic pressures in biological systems are usually estimated by indirect methods or by modeling.^[^ ^1^, ^3^, ^6^, ^10^ ^]^ In our previous work, the feasibility of spatiotemporal osmotic pressure measurements was demonstrated by resonance energy transfer (FRET) imaging with liposome‐based sensors loaded with suitable fluorescent dyes. Those sensors had a size of ≈1 µm and a sensing range of 0–0.3 MPa.^[^ ^11^ ^]^

**Figure 1:**
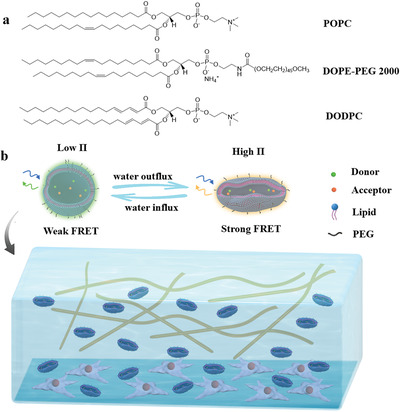
a) Chemical structures of the lipids used for the preparation of osmotic pressure sensors. b) Schematic illustration of the sensor working principle and the in situ osmotic pressure imaging in cell cultures.

The present work aims to extend the applicability of this sensor concept to biological tissues, through adjustment of the sensing range and biocompatible functionalization. To this end, novel kinds of FRET‐based liposomal sensors for the measurement of osmotic pressures are developed, which are loaded with two highly water‐soluble FRET dyes, for the in situ sensing of osmotic pressures in cell culturing media (**Figure** **1**). The semipermeable liposome membranes are formulated with naturally‐occurring phospholipids (POPC), lipid‐anchored hydrophilic polymers (DOPE‐PEG2000) for biocompatibilization and cross‐linkable phospholipids (DODPC) for stabilization. The FRET ratio, which is a measure of the FRET efficiency, varies as a result of changes in the intra‐liposomal dye concentration due to the extra‐liposomal osmotic pressure. Based on a known FRET ratio versus osmotic pressure curve, in situ osmotic pressure can be inferred from the FRET efficiency. The sensitivity and sensing range of the sensors can be readily adjusted by variation of the intra‐liposomal concentration of osmotically active species; here sodium chloride (NaCl) was used. The sensors are demonstrated to be suitable for the measurement of osmotic pressures in the pre‐osteoblast cell culture. The in situ imaging of osmotic pressures in cell culture is achieved with the help of FRET microscopy.

## 2 Results and Discussion

### 2.1 Preparation of Liposome‐Based Osmotic Pressure Sensors

In our previous work, POPC liposomes loaded with FRET donor (“D”) and acceptor (“A”) dyes in water were developed and applied for in situ spatiotemporal measurements of osmotic pressures by FRET imaging.^[^ ^11^ ^]^ In these liposomes, highly water‐soluble ATTO 488 and ATTO 542 were used as the donor and the acceptor, respectively. As a prerequisite for FRET, the fluorescence spectrum of ATTO 488 and the absorption spectrum of ATTO 542 in water show considerable overlap, in the range of 490–575 nm. For these liposomes, the FRET ratio exhibits signs of saturation above an osmotic pressure of П ≈ 0.3 MPa. Here, to extend the sensing range of the sensors toward physiological conditions, POPC liposomes were loaded with defined concentrations of NaCl, ranging from 0.05% mass fraction (8.6 mM) to 0.9% mass fraction (154 mM). In these sensors, the internal osmotic pressure induced by the salt opposes the sensor shrinkage and thus reduces the volumetric response of the sensors to external osmotic pressure, which results in extended sensing ranges. The average hydrodynamic diameters of these liposomes in water or corresponding NaCl solutions measured by dynamic light scattering (DLS) were all about 1 µm (**Table** **1**). The zeta potential increased with the increase of NaCl concentration from ≈−25 mV in water to near 0 in 0.9% NaCl, due to the screening effect of the salt (Table 1). In Figure S1 (Supporting Information), the size and zeta potential distributions are exemplified with Lip‐DA‐0.05 liposomes, where “DA” stands for the loading with donor and acceptor dyes and 0.05 stands for the (initial) intraliposomal NaCl concentration of 0.05%. For control, the fluorescence spectra of the donor, the acceptor, and the donor–acceptor 1:1 mixture were measured in the studied salt solutions. As seen in Figures S2 and S3 (Supporting Information), solvatochromic effects are negligible.

**Table 1:** Diameter and zeta potential of Lip‐DA liposomes, initial FRET ratio *R*_0_, slope, and sensing range for each liposome type in Figure 2

Liposome type	Diameter [nm]	Zeta potential [mV]	Initial *R*_0_ [%]	Slope [%/MPa]	Sensing range [MPa]
**Lip‐0**	1047 ± 37	−20.8± 0.2			
**Lip‐DA‐0**	1031 ± 32	−25.1 ± 0.6	125.3	374.4	0 – 0.3
**Lip‐DA‐0.05**	1052 ± 46	−4.9 ± 0.4	97.3	138.7	0.05 – 0.85
**Lip‐DA‐0.1**	1074 ± 63	−2.4 ± 0.2	86.6	106.2	0.08 – 1.2
**Lip‐DA‐0.2**	1045 ± 138	−2.2 ± 0.7	64.9	71.2	0.16 ‐
**Lip‐DA‐0.45**	1079 ± 82	−1.9 ± 0.2	62.0	25.7	0.35 ‐
**Lip‐DA‐0.9**	1108 ± 103	−1.1 ± 0.5	60.2	13.2	0.70 ‐

**Figure 2:**
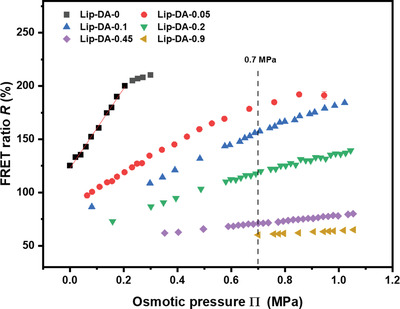
FRET ratio *R* obtained with Lip‐DA liposomes loaded with H_2_O, 0.05% NaCl, 0.1% NaCl, 0.2% NaCl, 0.45% NaCl and 0.9% NaCl and with a dye concentration of 50 µM (1:1 molar ratio) as a function of the external osmotic pressure П. The dashed vertical line indicates the physiological osmotic pressure around 0.7 MPa. The solid line shows exemplarily a linear fit to the data points for Lip‐DA‐0 within the linear range.

The osmotic responses of these salt‐loaded liposomes were determined with a series of extra‐liposomal NaCl solutions of known osmotic strengths. In order to quantify the FRET efficiency, the FRET ratio *R* was used, which is the ratio between the emission intensities at 562 nm (sensitized emission) and 520 nm (donor emission) in the recorded fluorescence spectra for an excitation wavelength of 458 nm. **Figure** **2** shows *R* of the intra‐liposomal dyes as a function of the osmotic pressure in the range of 0 ≤ П ≤ 1.05 MPa. The liposomes are expected to be vulnerable to osmotic pressure differences when the osmolality is lower outside than inside, where the liposomes can swell and lose their structural integrity. Therefore, the osmotic pressure equal to the initial one inside the liposomes was set as the starting point. The FRET ratio *R*_0_ at the starting point (i.e., at equal intra‐ and extraliposomal NaCl concentrations) decreases systematically with increasing NaCl concentration (see Table 1). For example, the FRET ratio of Lip‐DA‐0 liposomes (0 stands for zero intraliposomal NaCl concentration) is *R* ≈ 125%, while for Lip‐DA‐0.9 liposomes it is only *R* ≈ 60%. This tendency reflects that, due to ion screening, the electrostatic attraction between the oppositely charged donor and the acceptor fluorophores is weakened, leading to a larger average donor–acceptor distance, which in turn decreases the FRET efficiency.^[^ ^12^ ^]^ For all liposome types investigated, *R* increases monotonically with П, first approximately linearly, but then exhibits a weaker pressure dependence at higher П. This result is in line with our previous observations that the liposomes are more easily deformed from their initially spherical shape than in a partially deflated state resulting from the bending rigidity of the lipid bilayer.^[^ ^11^ ^]^ The initial slope of the *R*‐П curve at not‐too‐high osmotic pressure is a meaningful measure for the sensitivity of the sensors and decreases strongly and systematically with increasing intraliposomal NaCl concentration (Table 1). For example, the slope for Lip‐DA‐0 at low pressures (П ≲ 0.2 MPa) is as high as ≈ 370 %/MPa (see solid line in Figure 2), while for Lip‐DA‐0.9 at 0.70 ≲ П ≲ 1.05 MPa it is as low as ≈ 13 % per MPa. The change in the slope reflects that for a given difference between the extra‐ and intraliposomal osmotic pressure, weaker deflation is sufficient to compensate this difference—like the deformation mechanics of microcapsules^[^ ^13^ ^]^ and other kinds of vesicles^[^ ^14^ ^]^ under the effects of osmotic pressure, resulting in a smaller increase of the intra‐liposomal dye concentration. With that, the sensing range, which starts from the inherent osmotic pressure of the liposomes, can be readily adjusted toward and beyond the physiological osmotic pressure of around 0.7 MPa (value in human blood plasma). In Table 1 this is exemplified by the sensing range of 0.08–1.20 MPa for Lip‐DA‐0.1. However, as always, there is a trade‐off between the sensing range and the sensitivity, where the latter is closely related to the slope in the *R*‐П relation.

**Figure 3:**
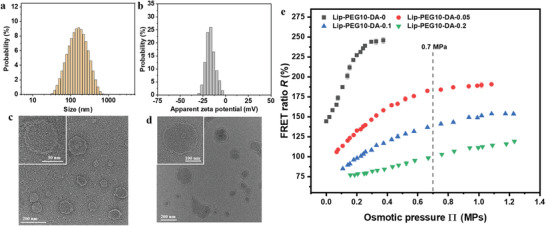
Characteristics and osmotic responses of Lip‐PEG10‐DA liposomes. Distributions of a) size and b) zeta potential of Lip‐PEG10‐DA‐0.05 liposomes in 0.05% NaCl as obtained by DLS and phase analysis light scattering (PALS), respectively. TEM images of c) Lip‐PEG10‐DA‐0 and d) Lip‐PEG10‐DA‐0.05 liposomes in dry state (inset, higher magnification) stained with 1% uranyl acetate. e) FRET ratio *R* obtained with Lip‐PEG10‐DA liposomes loaded with H_2_O, 0.05%, 0.1% and 0.2% NaCl and a dye concentration of 50 µM (1:1 molar ratio) as a function of the external osmotic pressure generated by various concentrations of NaCl.

In order to enhance biocompatibility, the POPC‐based sensor liposomes were doped with PEG lipids (DOPE‐PEG2000, see Figure 1a). The PEG chains with a contour length of about 13–17 nm (≈46 EG monomers) can endow the liposomes with steric stabilization and stealth properties, extend circulation half‐life, and reduce non‐specific protein binding ^[^ ^15^ ^]^ or cell adhesion, which is why PEGylation has been widely used for drug delivery, gene transfection as well as vaccine delivery.^[^ ^16^ ^]^ POPC liposomes doped with 5% (molar ratio) DOPE‐PEG2000, which were loaded with 50 µM ATTO 488 and 50 µM ATTO 542 in H_2_O or 0.05%, 0.1%, 0.2% (mass fraction) NaCl, denoted as Lip‐PEG5‐DA‐0/0.05/0.1/0.2, were first prepared. According to previous reports, the overlap threshold to the brush conformation regime is reached for a 4% molar ratio of lipids with a PEG2000 chain.^[^ ^17^ ^]^ DLS measurement results show that, although prepared under the same conditions as for the Lip‐DA liposomes (pore size of the extrusion membrane: 1.0 µm), the average hydrodynamic diameters of the Lip‐PEG5 liposomes (dye‐free liposomes doped with 5 mol% of PEG lipids) were in the range of only 250–350 nm (Table S1, Supporting Information). Under our experimental conditions, the incorporation of DOPE‐PEG2000 with its long hydrophilic headgroup can lower the energy barrier toward the formation of small liposomes with high curvature.^[^ ^18^ ^]^ However, liposome formation by extrusion is a complex process that depends on the rupture stability of the bilayers, their bending rigidity, and on their interactions with the extrusion pore material, among others. The presence of a PEG brush on the bilayer surface in general affects all of these aspects. It is therefore difficult to pinpoint which aspect ultimately governs the size of the resulting liposomes. Due to the presence of a negative charge in DOPE‐PEG2000, the zeta potentials of the Lip‐PEG5 liposomes are obviously more negative compared to un‐doped POPC liposomes, as shown in Table S1 (Supporting Information) and Table 1. Again, as a result of the salt screening effect, the zeta potential of the Lip‐PEG5‐DA liposomes becomes less negative with increasing NaCl concentration. Increasing the doping ratio of DOPE‐PEG2000 to 10% yielded liposomes of even smaller sizes (**Figure** **3**a; Table S2, Supporting Information) and more negative zeta potential (Figure 3b; Table S2, Supporting Information), as expected due to the reasons discussed above. Transmission electron microscopy (TEM) images show that the Lip‐PEG10‐DA liposomes are unilamellar (Figure 3c,d) with a bilayer of lipids.

The osmotic responses of the Lip‐PEG10‐DA liposomes (Figure 3e) are similar to those of Lip‐DA (Figure 2) and Lip‐PEG5‐DA liposomes (Figure S4, Supporting Information) with regards to sensitivity, sensing range as well as their dependence on the intraliposomal salt concentration. It should be noted that considering the preparation process and the different charge properties of the lipids, the concentration of the D and A dyes in the sensors may not be exactly the same as in the original bulk solutions. Several mechanisms could lead to an elevated dye concentration in the liposomes. For example, the concentration in the loading medium may be elevated due to evaporation effects during the hydration and freeze‐thaw cycles. And preferential interactions of the dyes with the liposome surface can result in some accumulation, which could even lead to an altered concentration ratio of acceptor and donor dyes in the liposomes. However, none of these possible sources for deviations are problematic because the calibration curves are made with the same sensors. In summary, osmotic pressure sensors with biocompatible surface functionalization and a suitable sensing/sensitivity range can be obtained, e.g. Lip‐PEG10‐DA‐0.05 liposomes for osmotic sensing under physiological conditions.

**Figure 4:**
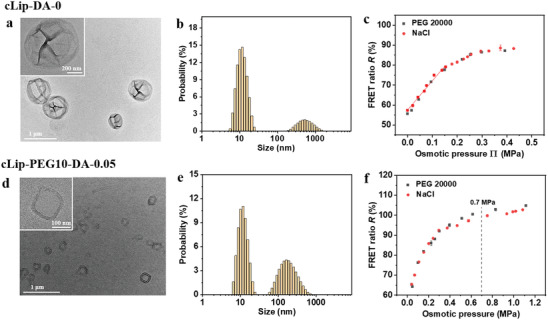
Characteristics and osmotic responses of crosslinked polymeric liposomes. a,d) TEM images of a) cLip‐DA‐0 and d) cLip‐PEG10‐DA‐0.05 liposomes in dry state (inset, higher magnification) stained with 1% uranyl acetate. b,e) Size distributions of b) cLip‐DA‐0 and e) cLip‐PEG10‐DA‐0.05 liposomes in water and 0.05% NaCl, respectively, in the presence of 0.3% Triton X‐100. c,f) FRET ratio *R* obtained with c) cLip‐DA‐0 and f) cLip‐PEG10‐DA‐0.05 liposomes loaded with a dye concentration of 25 µM (1:1 molar ratio) in H_2_O and 0.05% NaCl, respectively, as a function of the external osmotic pressure generated by various external concentrations of NaCl or PEG20000. The solid line in (c) shows exemplarily a linear fit to the data points for cLip‐DA‐0 in NaCl in the range of 0–0.15 MPa.

For applications in long‐term observations or in relatively harsh environments, osmotic pressure sensors with even higher stability may be required. For example, some microorganisms, especially bacteria, can produce biosurfactants^[^ ^19^ ^]^ that may destroy conventional liposomes. One of the most promising strategies to make the liposomal bilayers more stable is by chemically crosslinking polymerizable lipids within the bilayer.^[^ ^20^ ^]^ Polymerization of liposomal bilayers can be initiated by various methods such as radical initiators, UV and *γ*‐irradiation, among which UV irradiation is most commonly used because of its convenience. Liposomes of such covalently crosslinked lipid bilayers have been reported to be extremely stable in vitro and in vivo.^[^ ^21^ ^]^ Here, as a polymerizable lipid we used custom‐synthesized DODPC, which contains one diene group per acyl chain. To avoid photobleaching of the intraliposomal dyes, the polymerization of the monomeric DODPC lipids in liposomes was performed by the addition of the radical initiators 2,2’‐azobis(2‐methylpropionitrile) (AIBN) and 2,2’‐azobis(2‐methylpropionamidine) dihydrochloride (AAPD). The polymerization conversion for DODPC was analyzed by the spectral changes at 257 nm, corresponding to the UV absorption of the diene groups, and was found to be about 50–60% under our experimental conditions (Figure S5, Supporting Information). After removal of extra‐liposomal free dyes through rinsing and centrifugation, the crosslinked liposomes cLip‐DA‐0 were obtained, where “c” stands for the crosslinking and, as before, “0” indicates the absence of intra‐liposomal salt. TEM images (**Figure** **4**a) show that these liposomes form a number of multifold wrinkles in dry state due to the constrained fluidity of the crosslinked lipids, in contrast to Lip‐DA‐0 liposomes of similar size in our previous observations.^[^ ^11^ ^]^ The average hydrodynamic diameter in water, as measured by DLS (Figure S6a, Supporting Information) was found to be about 600 nm and the average zeta potential in water was ≈−32 mV (Figure S6b, Supporting Information). The impact of surfactants on the liposome morphology serves as a good parameter to evaluate the liposome stability. After the addition of 0.3% of the surfactant Triton X‐100, the size distribution of cLip‐DA‐0 liposomes did not change, as shown in Figure 4b (the peak around 10 nm is attributed to the Triton X‐100 micelles), suggesting no destruction or aggregation. In contrast, the Lip‐DA‐0 liposome peak disappeared immediately after Triton X‐100 addition (data not shown). The osmotic responses of the cLip‐DA‐0 liposomes were measured in NaCl and PEG20000 standard solutions. The FRET ratio at a given osmotic pressure was found to be consistent between the two solute types (Figure 4c). The FRET ratio increases with osmotic pressure approximately linearly in the range of 0–0.15 MPa with a slope of ≈150%/MPa, the slope is lower in the range 0.15–0.3 MPa (≈60% per MPa), and the FRET ratio starts saturating at around 0.3 MPa.

To adjust the properties of the crosslinked liposomes for applications under physiological conditions, cLip‐PEG10‐DA‐0.05 liposomes containing 10 mol% PEG lipids were fabricated. TEM on these liposomes in dry state shows a thick wall, which can be interpreted as a bilayer with a brush‐like corona of PEG (Figure 4d). The average hydrodynamic diameter was ≈ 250 nm and remained unchanged in the presence of 0.3% Triton X‐100, indicating the stability of the liposomes (Figure S7, Supporting Information; Figure 4e). The osmotic response plots are in an analogous shape to that of Lip‐PEG10‐DA‐0.05, of which the slope decreases progressively and the FRET ratio reaches saturation at about 1.2 MPa (Figure 4f). This behavior suggests that also with the cross‐linked liposomes the sensing range can be tuned with the internal salt concentration. It should however be noted that crosslinked liposomes are required only under harsh conditions and rely on custom‐synthesized cross‐linkable lipids for their preparation. For the following cell culture experiments, the non‐crosslinked liposomes were therefore used.

### 2.2 Biocompatibility and Osmotic Pressure Sensing under Cell Culture Conditions

**Figure 5:**
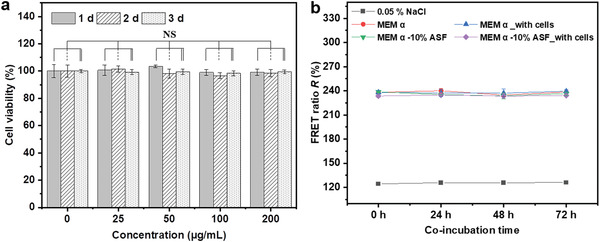
Cytotoxicity and sensor functionality in cell culture media. a) Viability of MC3T3‐E1 cells after co‐incubation with 25, 50, 100, and 200 µg mL^−1^ Lip‐PEG10‐DA‐0.05 liposomes for 1, 2 and 3 d, respectively. b) FRET ratio obtained with Lip‐PEG10‐DA‐0.05 sensors loaded with a dye concentration of 75 µM (1:1 molar ratio) after incubation in NaCl or MEM *α* cell culture media supplemented with 10% ASF with/without MC3T3‐E1 cells for 24, 48, and 72 h at 37 °C. Data in (a) are expressed as the mean ± standard deviation (SD), *n* = 5. NS indicates no significant difference at a level of *p* < 0.05.

As a preliminary step toward in situ sensing of osmotic pressures in tissues, the application of the developed liposomal sensors in the in vitro cell culture systems was explored with MC3T3‐E1 pre‐osteoblast cells which have been demonstrated to synthesize a collagen‐rich ECM during in vitro tissue cultures and to respond to mechanical cues^[^ ^22^ ^]^ as well as curvature^[^ ^23^ ^]^ and NIH3T3 fibroblast cells. To this end, the Lip‐PEG10‐DA‐0.05 liposomes were utilized, which were prepared under sterile conditions and with formulation principles similar to those widely used for liposomal drug delivery systems.^[^ ^24^ ^]^ The biological applicability of the liposomes was initially evaluated i) by measuring the toxicity to MC3T3‐E1 and NIH3T3 cells using the EZ4U cell proliferation and cytotoxicity assay and ii) by testing the longevity of sensor functionality under cell culture conditions. EZ4U measures the ability of living cells to reduce a colorless tetrazolium salt to an orange water‐soluble formazan derivative by mitochondrial dehydrogenases.^[^ ^25^ ^]^ As shown in **Figure** **5**a and Figure S8 (Supporting Information), the Lip‐PEG10‐DA‐0.05 liposomes had no detectable influence on cytoviability when they were incubated with MC3T3‐E1 or NIH3T3 cells for 24, 48, and 72 h at a concentration of 25, 50, 100, and 200 µg mL^−1^ at 37 °C. The negligible cytotoxicity of these liposome sensors clearly favors their applications in tissues. In the next step, the osmotic response of the Lip‐PEG10‐DA‐0.05 sensors was investigated in diluted and undiluted Minimum Essential Medium *α* (MEM *α*) medium, which is commonly used for MC3T3‐E1 cell cultures. The samples were measured at room temperature (r.t.) immediately after addition of the sensors to the solutions. The osmotic pressure was found to increase approximately linearly with concentration of the medium according to the results measured with a freezing point osmometer (Figure S9, Supporting Information). As shown in Figure S10 (Supporting Information), the FRET ratios as a function of osmotic pressure in MEM *α*, in MEM *α* supplemented with 10% fetal bovine serum (FBS), and in the dilutions coincide well with those observed in NaCl solutions, indicating the functionality of the sensors in the cell culture media under the experimental conditions. In order to examine whether the sensors remain functional over longer time scales and under cell culture conditions, time‐dependent measurements were conducted. At r.t. and without cells, the FRET ratios of the sensors in 0.05% NaCl (≈0.05 MPa), 0.9% NaCl (≈0.7 MPa), MEM *α* (≈0.7 MPa), and 2× diluted MEM *α* (≈0.35 MPa) remained virtually constant after incubation of the sensors in these solutions for 0, 1, 3, 6, and 30 h (Figure S11a, Supporting Information). However, in MEM *α* with 10% FBS (≈0.7 MPa) and 2× diluted MEM *α* with 10% FBS (≈ 0.35 MPa), the FRET ratios decreased significantly after 30 h, an observation to which we get back further below. Under cell culture conditions (37 °C), whether co‐incubated with MC3T3‐E1 cells or not, the Lip‐PEG10‐DA‐0.05 sensors were still functional in 0.05% NaCl, 0.9% NaCl and MEM *α* after 24, 48, and 72 h (Figure S11b, Supporting Information). Therefore, it can be inferred that the sensors are stable and work well in the MEM *α* medium over a relatively long time, and that the cells do not damage the liposomes or influence the effectivity of the sensors under the experimental conditions. However, again, the FRET ratios in MEM *α* with 10% FBS dropped over time both in the presence and absence of cells. It is reported that lipoproteins in the plasma, which is a biochemical assembly whose primary function is to transport hydrophobic lipid molecules in water, can interact with the liposomes and cause changes on the structure of liposomes surface (lipid transfers/depletion) with the reduction of their colloidal stability leading to the leakiness of the liposomes.^[^ ^26^ ^]^ When the lipoprotein‐to‐phospholipid ratio is low enough, the liposomes remain intact. Therefore, the decrease of FRET ratio in MEM *α*‐10% FBS can be attributed to the leakage of dyes as a result of the lipoprotein effect on the liposomes.

This problem can be solved by using lipoprotein‐deficient or synthetic serum in in vitro cell culture systems. Strategies to control liposomal structural stability can also be used, such as working with crosslinked liposomes as the ones introduced above. In the present study we have focused on the non‐crosslinked liposomes and consequently used synthetic serum. The time‐dependent functionality of the sensors in the cell culture system with MEM *α* supplemented with artificial serum (AS) was then studied. Two kinds of commercial artificial serum – FastGro (ASF) and Panexin (ASP) were used. The sensors for this purpose were loaded with 75 µM donor and acceptor dyes in 0.05% NaCl, which exhibit a suitable sensitivity in the desired osmotic pressure range (0.05–1.2 MPa; Figure S12, Supporting Information). The osmotic responses of these sensors in MEM *α*, MEM *α* with 10% ASF and their dilutions again conform to those in NaCl solutions. After co‐incubation with MC3T3‐E1 cells in 0.05% NaCl, 0.9% NaCl, PBS (phosphate‐buffered saline), MEM *α* and MEM *α* with10% ASF for 24, 48 and 72 h, the FRET ratios of the sensors remained virtually unchanged (Figure 5b; Figure S13a, Supporting Information), confirming the long‐time functionality of the sensors in these cell‐media systems. The same holds for media supplemented with another artificial serum (MEM *α* with10% ASP) with/without cells for 24, 48, and 72 h (Figure S13b, Supporting Information). There are many interesting problems on the time scales of hours to days, so we consider a stability proof over 3 days sufficient for the moment. Future studies could be aimed at optimizing the sensor design for long‐term observations.

### 2.3 In Situ Osmotic Pressure Imaging in Cell Cultures

**Figure 6:**
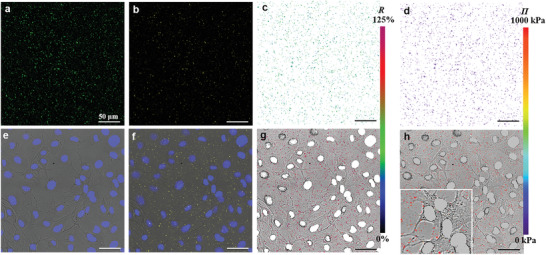
Application of Lip‐PEG10‐DA‐0.05 sensors for osmotic pressure imaging in cell culture. Confocal laser scanning microscopy (CLSM) images of sensors (125 µg mL^−1^)/cells in a,b) 0.05% NaCl and e,f) MEM *α*‐10% ASF with MC3T3‐E1 cells. The green fluorescence in (a) and yellow fluorescence in (b,f) represent the donor emission signal (Ex 458 nm, Em 468–538 nm) and the sensitized acceptor emission signal (Ex 458 nm, Em 571–700 nm), respectively. (e) shows the live MC3T3‐E1 cells with the nuclei stained with Hoechst 33342 (Ex 405 nm, Em 415–450 nm). (f) demonstrates the functioning of the sensors around the cells. c,g) FRET ratio and d,h) osmotic pressure mapping with the sensors in 0.05% NaCl (c,d) and MEM *α*‐10% ASF with MC3T3‐E1 cells (g,h) (inset, higher magnification), respectively. The green (c) and magenta (g) dots indicate relatively low FRET ratio in 0.05% NaCl and high FRET ratio in the medium, respectively. The purple (d) and red (h) dots indicate relatively low osmotic pressure in 0.05% NaCl and high osmotic pressure in the medium, respectively.

On the basis of the ex situ measurements, the in situ sensing of osmotic pressures in a cell culture system with the sensors was further explored. As in our previous study, ^[^ ^11^ ^]^ confocal laser scanning microscopy (CLSM) was utilized for sensitized emission FRET imaging (excitation wavelength 458 nm). As shown in **Figure** **6** and Figures S14 and S15 (Supporting Information), well‐dispersed individual liposomes can be observed in 0.05% NaCl (Figure 6a,b), in the culture medium MEM *α*‐10% ASF (Figure S14a–c, Supporting Information), and in MEM *α*‐10% ASF with MC3T3‐E1 cells (Figure 6e,f; Figure S15a–c, Supporting Information) on glass‐bottomed cell culture dishes. The exhibited donor emission (Figure 6a; Figures S14a and S15a, Supporting Information), sensitized acceptor emission (Figure 6b,f; Figures S14b and S15b, Supporting Information), and direct acceptor emission signals (Figures S14c and S15c, Supporting Information; excitation wavelength 561 nm) visualize the presence of the donor, the FRET effect, and the acceptor in the sensors, respectively. The lower fluorescence intensities of the donor signal (Figures S14a and S15a, Supporting Information) and higher intensities of the sensitized acceptor emission signal (Figures S14b and S15b, Supporting Information) in the MEM *α*‐10% ASF qualitatively confirm the stronger FRET effect associated with the higher osmotic pressure, compared with that in 0.05% NaCl (Figure 6a,b). The FRET efficiency is then quantified in the form of the FRET ratio *R* between the sensitized acceptor emission and the donor emission for each pixel. Figure 6c,g and Figures S14d and S15d (Supporting Information) show FRET ratio images of sensors in 0.05% NaCl (Figure 6c), MEM *α*‐10%ASF (Figure S14d, Supporting Information), and MEM *α*‐10%ASF with MC3T3‐E1 cells (Figure 6g; Figure S15d, Supporting Information), in which the osmotic pressure difference between the 0.05% NaCl and the media as well as the consistence between the media with and without cells are explicitly reflected by the FRET ratios. For quantitative analysis and osmotic pressure mapping, the corresponding *R‐*П calibration curve was obtained by using the average FRET ratio of the sensor‐containing pixels selected by segmentation (pixels with intensities below the noise threshold were excluded from segmented images) in MEM *α*‐10%ASF and the dilutions, or MEM *α*‐10%ASF with added NaCl. As shown in Figure S16 (Supporting Information), *R* increases systematically with increasing osmotic pressure, from *R* ≈ 43% at 0.05 MPa to *R* ≈ 80% at 0.93 MPa. It should be noted that the collection and quantification methods are different for CLSM and spectrofluorometer. For example, for CLSM, a 458 nm laser is used for excitation and signals detected over a range of wavelengths were used for the calculation of *R*, while with the spectrofluorometer, a broader band of light (458 nm, bandwidth 3.5 nm) was used for excitation and the spectral peak intensities signals are used to define the emission signals (see Supporting Information for details). As a result, the absolute FRET ratio values from the fluorescence microscopy are systematically different from those obtained by fluorescence spectroscopy. Still, the shapes of the calibration curves, which are governed by the sensor properties, are consistent between the two techniques (Figures S12 and S16, Supporting Information), and the differences are accounted for by the different calibration curves. Figure 6d,h and Figures S14e and S15e (Supporting Information) illustrate the successful application of the sensors for in situ osmotic pressure mapping with the *R‐*П calibration curve (Figure S16, Supporting Information). In the osmotic pressure mapping image, the osmotic pressures around the cells can be measured with a spatial resolution of ≈ 0.2 µm (Figure 6h; Figure S15e, Supporting Information). In a system with continuous liquid, the osmotic pressure distribution should also be continuous. The osmotic pressure imaging above is pixelated (Figure 6d,h; Figures S14e and S15e, Supporting Information) because of the imaging method, i.e., pixels with a large signal correspond to regions containing one or several liposomes, while regions with low signal‐to‐noise ratio (not containing liposomes) were excluded from segmented images. To demonstrate their applicability in in situ measurements of dynamic processes, the sensors were used to monitor the osmotic pressure while changing the culture medium at lower magnification (eyepiece 100×). As shown in Figures S17, S18 and Videos S1–S5 (Supporting Information), the sensors report an increase in the osmotic pressure (with the *R‐*П calibration curve Figure S19, Supporting Information) around the cultured cells when the culture medium is exchanged with one that has higher osmolarity.

In this application, it is an important concern whether the sensors can be internalized by the cells. Therefore, the cellular uptake behavior of the sensors was quantitatively evaluated with flow cytometry, which measures the fluorescence of single cells. Sensor uptake would thus increase the fluorescence of the cells (see Supporting Information for the details). After co‐incubation of the sensors (125 µg mL^−1^) with MC3T3‐E1 or NIH3T3 cells for 24 h, the ratio of cells that internalized sensors is immeasurably low because the average fluorescence intensity per cell changes very little (Figure S20a–d, Supporting Information) in comparison with the control group in which the cells were incubated without sensors but otherwise under the same conditions (Figure S21a–d, Supporting Information). The negligible cellular uptake of the sensors is beneficial for their applications in the environment around cells, such as in media, hydrogels, or tissues. If, on the other hand, the osmotic pressure inside cell milieu is of interest, the sensors could in principle be administered directly into the cells, by microinjection. However, whether or not cell function would be affected by this procedure will first have to be tested.

Having established the applicability of such sensors for the in situ spatiotemporal imaging of osmotic pressures already in our previous work,^[^ ^11^ ^]^ the present work further demonstrates the compatibility and application of such sensors in life cell media and tissues. In summary, we were able to demonstrate the feasibility of in situ measurements of osmotic pressures in cell cultures and tissues. Biological systems are usually dynamic as biological entities are constantly interacting with the environment. Given the observed stability of the sensors, the spatiotemporal evolution of osmotic pressures in tissues could also be monitored over longer periods. Finally, the method can be readily extended to 3D systems and thus to various biological settings and contexts. For example, it would be interesting to explore the length and time scales of osmotic pressure gradients inside and around cell colonies, noting that reliable theoretical predictions are still lacking. In future work, we intend to investigate osmotic pressure on the supra‐cellular length scale using a micro‐tissue system established in our lab.^[^ ^23^ ^]^

## 3 Conclusions

Osmotic pressure sensors based on dye‐loaded liposomes with adjustable sensitivity and sensing range have been developed for the purpose of in situ applications in bio‐relevant systems. This strategy allows producing sensors optimized for a wide range of osmotic pressures with good sensitivity, including the physiological conditions. Sensors based on PEGylated liposomes with steric stabilization and stealth property were prepared to increase the stability and minimize the undesirable biological interactions. Crosslinked liposome sensors were developed for applications in relatively harsh environments (e.g., in the presence of surfactants) and also have the potential for long‐term measurements. Moreover, as preliminary steps toward in situ sensing of osmotic pressures in biological tissues, the functionality of the sensors in pre‐osteoblast cell culture systems has been demonstrated. On this basis, with the help of FRET microscopy, the in situ spatially‐resolved sensing of osmotic pressures in a cell culture system was demonstrated. This study may pave a solid road toward the in situ spatiotemporal imaging of osmotic pressures in biological in vitro systems.

## 4 Experimental Section

### Materials

1‐palmitoyl‐2‐oleoyl‐glycero‐3‐phosphocholine (POPC) and 1,2‐dioleoyl‐sn‐glycero‐3‐phosphoethanolamine‐*N*‐[methoxy(polyethylene glycol)‐2000] (ammonium salt) (DOPE‐PEG2000) were purchased from Avanti Polar Lipids (Alabaster, USA). 1,2‐di(2,4‐octadecadienoyl)‐glycero‐3‐phosphocholine (DODPC) was custom‐synthesized by ShoChem Co., Ltd (Shanghai, China). The purity of DODPC was confirmed by thin‐layer chromatography (Merk, silica gel 60 matrix) with chloroform/methanol/water (65/25/4, by volume). Samples showing a single spot with an *R*_f_ value of around 0.4 were used for the experiments. ATTO 488 carboxy and ATTO 542 carboxy were purchased from ATTO‐TEC GmbH (Siegen, Germany). NaCl was purchased from neoLab Migge (Heidelberg, Germany). AIBN, Sephadex G‐50, and Triton X‐100 were purchased from Sigma–Aldrich (Steinheim, Germany). AAPD was purchased from Acros Organics (Geel, Belgium). Chloroform, methanol and ethanol were purchased from Merck KGaA (Darmstadt, Germany). MEM *α* without phenol red was purchased from Thermo Fisher Scientific (Waltham, USA). FBS was purchased from PAA Laboratories (Linz, Austria). Artificial serum FastGro (ASF) and Panexin (AFP) were purchased from Fisher Scientific (Schwerte, Germany) and PAN‐Biotech (Passau, Germany), respectively. Hoechst 33342 was purchased from Invitrogen (Carlsbad, USA). The water used in all experiments was ultrapure water (18.2 MΩ). AIBN and AAPD were purified twice by recrystallization from ethanol and water, respectively. All chemicals used in the experiments were of pharmaceutical standard and analytical grade.

### Characterization

Size/zeta potential was measured with a size/ zeta potential analyzer (Zetasizer Nano‐ZS, Malvern) equipped with a 632.8 nm He‐Ne laser at room temperature (25 °C). Each value was averaged from three parallel measurements. UV–vis absorption spectra were recorded with an Analytik Jena UV‐Vis Specord 210 Plus spectrophotometer. Fluorescence emission spectra were recorded with a Horiba Fluoromax4 spectrofluorometer. For UV–vis spectra, fluorescence spectra and zeta potential/size measurements, the sample solutions were used directly. TEM images were recorded on a JEOL COM instrument at an acceleration voltage of 200 kV. Samples were prepared by placing a drop of the liposome suspension onto a carbon film‐coated copper grid and then the liposomes were stained with 1% uranyl acetate. CLSM: The liposome suspension in NaCl solutions or MEM *α* media on a 20 mm cell culture dish with a glass bottom was observed with a Leica TCS SP8 system (40×/1.3 NA oil immersion objective or 10×/0.3 NA water immersion objective using commercial software). Flow cytometry: The average fluorescence intensity per cell and the ratio of cells with fluorescence were measured with flow cytometry (FACS Calibur, BD).

### Emission Spectra Measurement of Bulk Dye Solutions

The emission spectra of ATTO 488 carboxy, ATTO 542 carboxy and their 1:1 mixture solutions (1 µM) in water or 0.05%,0.1%,0.2%, 0.45% or 0.9% NaCl were measured with a Horiba Fluoromax4 spectrofluorometer. The excitation wavelength was set at 458 nm, and bandwidths for the excitation and emission path were both 2.5 nm. Integration time was 0.1 s.

### Preparation of Lip‐DA and Lip‐PEG‐DA Liposomes

The liposomes were prepared by the extrusion method using a Mini‐Extruder (Avanti Polar Lipids Inc.). Removal of extra‐liposomal free dyes was achieved via rinsing and centrifugation or gel permeation chromatography. 5 mg POPC or POPC with DOPE‐PEG2000 lipids were dissolved in 0.5 mL chloroform and were evaporated by passing a gentle stream of nitrogen over the sample, followed by drying under vacuum overnight. The lipid film was hydrated with 0.5 mL mixture solution of ATTO 488 carboxy (50 µM) and ATTO 542 carboxy (50 µM) in water or NaCl solution (0.05%, 0.1%, 0.2%, 0.45% or 0.9%) for 1 h at 30 °C. Then the mixture was vortexed and was subjected to 5 freeze‐thaw cycles by alternately placing the sample vial in a liquid nitrogen bath and warm water bath. The suspension was extruded through a polycarbonate membrane (pore size 1.0 µm; Avanti Polar Lipids, 610 010) 21 times at 30 °C. The liposomes were incubated at 4 °C overnight to minimize structural defects of the molecular packing in the liposomes. For Lip‐DA liposomes, free dyes were removed by washing with centrifugal filters (Amicon Ultra‐2 100K) (10 000 g, 10 min). For Lip‐PEG‐DA liposomes, free dyes were removed by gel permeation chromatography on Sephadex G‐50.

### Preparation of cLip‐DA and cLip‐PEG10‐DA Liposomes

5 mg DODPC or DODPC with DOPE‐PEG2000 lipids dissolved in 0.5 mL chloroform were mixed with 110 µL 0.5 mg mL^−1^ AIBN/chloroform (5 mol% to the monomeric lipids). The mixture was evaporated by passing a gentle stream of nitrogen over the sample, followed by drying under vacuum for 2 h. The lipid film was hydrated under a nitrogen atmosphere with 1 mL mixture solution of ATTO 488 carboxy (25 µM) and ATTO 542 carboxy (25 µM) in degassed water or NaCl solution (0.05%) for 1 h at r.t that was above the phase transition temperature 18 °C of DODPC.^[^ ^20c^ ^]^ Then the mixture under a nitrogen atmosphere was vortexed and was subjected to 5 freeze‐thaw cycles by alternately placing the sample vial in a liquid nitrogen bath and room‐temperature water bath. Then 86 µL 1 mg mL^−1^ AAPD/water (5 mol% to the monomeric lipids) was added to the suspension. The suspension was extruded through a polycarbonate membrane (pore size 1.0 µm ; Avanti Polar Lipids, 610 010) 21 times at r.t. The liposome suspension was incubated at 4 °C for 2 h to minimize structural defects of the molecular packing in the liposomes. In a 25 mL Schlenk tube, the liposome suspension was degassed in vacuum and then the tube was backfilled with nitrogen, and sealed after three degassing‐nitrogen filling cycles. In the liposomes, AIBN and AAPD were introduced into a hydrophobic region and an aqueous phase, respectively. The liposomes were polymerized for 12 h at 60 °C. The polymerization conversion for DODPC was analyzed by the spectral changes at 257 nm, corresponding to the UV absorption of the diene groups. Free dyes were removed by washing with centrifugal filters (Amicon Ultra‐2 100K) (10 000 g, 10 min).

### Osmotic Strength Measurement of Standard Solutions

The NaCl or PEG 20 000 standard solutions were prepared with the weighing method at room temperature. The osmolalities of NaCl solutions and MEM *α* (dilutions) were determined from freezing point depression using an OSMOMAT 3000 Osmometer (Gonotec GmbH). Standards (0, 300, and 850 mOsm kg^−1^) were analyzed prior to samples which were measured at least in triplicate. PEG 20 000 solution osmolalities were determined from vapor pressure depression using a VAPRO MODEL 5600 Osmometer (ELITech Group, Inc.). Standards (100, 290, and 1000 mOsm kg^−1^) were analyzed prior to samples which were measured at least five times.

### Calibration Curves Measurement of Sensors in Standard Solutions with Spectrofluorometer

The liposome sensors were dispersed in water or the corresponding NaCl solution at a concentration of 2.5 mg mL^−1^. Then 5 µL suspension was added to 100 µL standard solutions and the emission spectra were recorded with spectrofluorometer (Horiba MC Fluoromax4). The excitation wavelength was set at 458 nm and the emission spectra were recorded at 480–640 nm. Bandwidths for the excitation and emission path were both 3.5 nm. Integration time was 0.1 s. The osmotic pressure of the mixture was corrected by calculation according to the osmotic pressure‐mass fraction calibration curve of the standard solutions. The FRET ratio *R* (F_562_/F_520_) was calculated and its variation with varying osmotic pressure was analyzed to study the osmotic response of liposomes in different solutions. For measurements in MEM *α* (dilutions), background signals were measured under the same conditions: 5 µL water or the corresponding NaCl solution was added to 100 µL standard solutions and the emission spectra were recorded as above. The FRET ratios were calculated by using corresponding fluorescence signals of the liposome sensors obtained by subtracting the background from the total signals.

### Time‐Dependent Measurement of the Sensors in Cell Culture Media and NaCl Solutions

50 µL liposome sensor suspension in 0.05% NaCl solution (2.5 mg mL^−1^) was added to 1 mL MEM *α* (dilutions) or NaCl solutions. At desired time points, 100 µL was taken for emission spectra measurement with spectrofluorometer. Background signals of the MEM *α* (dilutions) were measured under the same conditions: 50 water or corresponding NaCl solution was added to 1 mL MEM *α* (dilutions). At desired time points, 100 µL was taken and the emission spectra were recorded. The FRET ratios *R* (F_562_/F_520_) were calculated by using corresponding fluorescence signals of the liposome sensors obtained by subtracting the background from the total signals.

### Acquisition and Analysis of Confocal Microscopy Images

The sensor suspensions were imaged on a Leica TCS SP8 confocal laser scanning microscope for FRET evaluation using a 40× oil immersion objective or 10× water immersion objective. CLSM allows for image acquisition of different channels pixel‐per‐pixel practically at the same time. The settings of the three channels were as follows: donor channel, excitation at 458 nm, detection at 468–538 nm; FRET channel, excitation at 458 nm, detection at 571–700 nm; acceptor channel, excitation at 561 nm, detection at 571–700 nm. All settings (HyD detector parameters (voltage, offset), pixel dwell time, laser power, electronic zoom and pinhole) were kept constant across all FRET experiments. The image sets of the samples were acquired for FRET evaluation. In the liposomes, the donor and acceptor had a fixed 1:1 stoichiometry. Therefore, the ratio of FRET signal (sensitized acceptor emission) intensity F_FRET_ to the donor signal intensity F_donor_ was adopted as the index of FRET efficiency (*R*). The pixel‐by‐pixel FRET ratio images were obtained by processing the raw image data sets with the Fiji software.^[^ ^27^ ^]^

### Calibration Curves Measurement of Sensors in MEM *α* with Confocal Microscopy

The Lip‐PEG10‐DA‐0.05 sensors loaded with a dye concentration of 75 µM (1:1 molar ratio) were dispersed in 0.05% NaCl at a concentration of 10 mg mL^−1^. Then 1 µL suspension was added to 80 µL 0.05% NaCl, MEM *α*‐10%ASF or dilutions. The osmotic pressure of the mixture was corrected by calculating according to the osmotic pressure‐mass fraction calibration curve. A drop of the obtained suspension was placed on a 20 mm cell culture dish with a glass bottom. The image sets for the donor, FRET and acceptor channels were acquired with confocal microscopy (Leica TCS SP8, 40×/NA 1.3 oil immersion objective), as stated above. For each sample, at least three image sets were recorded in different areas of the sensor suspension drop. NaCl or media solutions without sensors as control samples were measured under the same conditions. For the calculation of the average FRET ratio of the samples, the following steps were carried out. Using Fiji software, by segmentation the sensor pixels were selected and used for the calculation of FRET ratio. Segmentation involved finding the maximum value pixel of each channel from the images of corresponding control groups that contained no sensors, and then setting a threshold just above this pixel intensity (i.e., the noise threshold). Pixels with intensities below the noise threshold were excluded from segmented images. The average FRET ratio was obtained as follows: the sum fluorescence intensity of sensor‐containing pixels of a region of interest (150 µm × 150 µm in the center of the image) in a donor channel or FRET channel image was calculated; then the ratio of FRET sum intensity F_FRET_ to the donor sum intensity F_donor_ was calculated as the FRET ratio (*R*). Then the average FRET ratio of the parallel image sets was calculated and was plotted versus osmotic pressure to calibrate the osmotic response of sensors in 0.05% NaCl, MEM *α*‐10%ASF or dilutions.

### Cell Culture

Murine preosteoblastic cells MC3T3‐E1 were provided by the Ludwig Boltzmann Institute of Osteology (Vienna, Austria). The MC3T3‐E1 cells were cultured in MEM *α* (Sigma–Aldrich, St. Louis, MO) with D‐glucose (4500 mg L^−1^; Sigma–Aldrich, Germany). The medium was supplemented with 5% FBS (PAA Laboratories, Linz, Austria), ascorbic acid (50 µg mL^−1^; Sigma–Aldrich, St. Louis, MO), and 0.1% gentamicin (Sigma–Aldrich, Steinheim, Germany). Murine embryonic fibroblasts NIH3T3 were purchased from DMSZ. The NIH3T3 cells were cultured in Dulbeco's modified eagle's medium (DMEM) (Sigma–Aldrich, St. Louis, MO) supplemented with 5% FBS and 0.1% gentamicin. The cells were incubated in an incubator at 37 °C supplied with 5% CO_2_ and 100% humidity.

### Cytotoxicity Assay

Cell activity was determined with EZ4U assay (Biomedica, Vienna, Austria) as an indicator of cytotoxicity. MC3T3‐E1 or NIH3T3 cells were seeded at a density of 5×10^3^ cells per well on 96‐well plates and were incubated for 24 h. Then the cells were incubated in incubator with the Lip‐PEG10‐DA‐0.05 liposomes at a concentration of 25, 50, 100 and 200 µg mL^−1^ for 24, 48 or 72 h. At desired time points, the media containing liposomes were removed, followed by washing three times with PBS to remove the free liposomes. EZ4U assay reagents were prepared as follows: dissolve one vial of substrate (SUB) in 2.5 mL activator (ACT) and pre‐warm this solution to 37 °C prior to addition. 180 µL media and 20 µL SUB‐ACT solution were added into each well. After 3 h incubation at 37 °C, mix by tipping the plate and transfer 100 µL from each well to a new 96‐well plate. The absorbance at 450 nm was measured by a microplate reader (Cytation 5, BioTek). Cell activity was expressed as the ratio of absorbance of the experimental groups to that of the control group in which the cells were incubated with cell culture medium only. Data were expressed as average ± SD (*n* = 5).

### Time‐Dependent Measurement of the Sensors after Co‐Incubation with Cells

MC3T3‐E1 cells were seeded at a density of 5×10^3^ cells per well on 96‐well plates and were incubated for 24 h. Then the cells were incubated in incubator with the Lip‐PEG10‐DA‐0.05 liposomes at a concentration of 125 µg mL^−1^ in MEM *α*, MEM *α*‐10% FBS, MEM *α*‐10%AS, PBS and 0.9% NaCl for 24, 48 or 72 h. At desired time points, the media containing liposomes were taken and measured with spectrofluorometer. The FRET ratios were calculated by using corresponding fluorescence signals of the liposome sensors obtained by subtracting the background from the total signals. Data were expressed as average ± SD (*n* = 3).

### In Situ Sensing of Osmotic Pressures in Cell System by FRET Imaging with the Sensors

The in situ sensing of osmotic pressures in cell system with the sensors was conducted by using the confocal microscopy (Leica TCS SP8, 40×/NA 1.3 oil immersion objective). The MC3T3‐E1 cells were seeded at a density of 8×10^4^ on a 20 mm cell culture dish with a glass bottom, and were cultured for 24 h. Then the cell culture media were removed and 500 µL 0.005 mg mL^−1^ Hoechst 33 342 in PBS was added. After incubation in the incubator for 8 min, the staining solution was removed and the cells were washed three times with PBS. The Lip‐PEG10‐DA‐0.05 sensors loaded with a dye concentration of 75 µM (1:1 molar ratio) were dispersed in 0.05% NaCl at a concentration of 10 mg mL^−1^. Then 5 µL suspension was added to 400 µL MEM *α*‐10%ASF. The osmotic pressure of the mixture was corrected by calculating according to the osmotic pressure‐mass fraction calibration curve. The suspension of sensors in the media was added to the cells and the sample was observed with confocal microscopy (Leica TCS SP8, 40×/NA 1.3 oil immersion objective). The image sets for the donor, FRET and acceptor channels were acquired, as stated above. The channels for imaging cells were as follows: transmission channel, 561 nm laser line; Hoechst 33 342 channel, excitation at 405 nm, detection at 415–450 nm. For each sample, at least five image sets were recorded in different areas. For the mapping of FRET ratio of the samples, the following steps were carried out. Using Fiji software, by segmentation the sensor pixels were selected as regions of interest and used for the calculation of pixel‐by‐pixel FRET ratio. After excluding outliers, the FRET mapping was achieved. By taking advantage of the *R‐*П calibration curve, osmotic pressures could be quantified spatially around the cells on the FRET image.

### In Situ Monitoring of Osmotic Pressures When Changing Cell Culture Media

The in situ sensing of osmotic pressures in cell system with the sensors was conducted by using the confocal microscopy (Leica TCS SP8, 10×/NA 0.3 water immersion objective). The MC3T3‐E1 cells were seeded at a density of 1×10^5^ on a 20 mm cell culture dish with a glass bottom, and were cultured for 24 h. The cell culture medium with phenol red was removed and the cells were washed three times with PBS. Then the cells were imaged in media without phenol red. 800 µL medium, medium‐0.3%NaCl, sensors/medium (125 µg mL^−1^) and sensors/medium‐0.3%NaCl (125 µg mL^−1^) were added sequentially. The image sets were acquired by time‐lapse imaging (time interval 21 s, duration 24 min). The suspensions of sensors in medium or medium‐0.3%NaCl were prepared as follows. The Lip‐PEG10‐DA‐0.05 sensors loaded with a dye concentration of 100 µM (1:1 molar ratio) were dispersed in 0.05% NaCl at a concentration of 10 mg mL^−1^. Then 10 µL this suspension was added to 800 µL MEM *α*‐10%ASP medium or medium‐0.3%NaCl. The osmotic pressure of the mixture was corrected by calculating according to the osmotic pressure‐mass fraction calibration curve. The mixture was added to the cells and was imaged immediately with confocal microscopy (Leica TCS SP8, 10×/NA 0.3 water immersion objective). The image sets for the donor, FRET and acceptor channels were acquired, as stated above. The channel for imaging cells was the transmission channel (561 nm laser line). For the mapping of FRET ratio of the samples, the following steps were carried out. The images of media only with cells were used as background. Using Fiji software, backgrounds were subtracted from the images of sensors/media with cells. By segmentation, the pixels of low fluorescence intensity and saturated pixels were excluded. Then the pixel‐by‐pixel FRET ratio was calculated and the FRET mapping during the in situ monitoring of medium changing was achieved. The *R‐*П calibration curve of the sensors in MEM *α*‐10%ASP or dilutions or (MEM *α*‐10%ASP)‐0.3%NaCl was obtained using the same acquisition settings of the images as above and the same calculation method as in “*Calibration curves measurement of sensors in MEM α with confocal microscopy*”. By taking advantage of the *R‐*П calibration curve, osmotic pressures can be quantified spatially around the cells on the FRET image.

### Evaluation of Cellular Uptake of the Sensors

The cellular uptake of the Lip‐PEG10‐DA‐0.05 sensors loaded with a dye concentration of 75 µm (1:1 molar ratio) was analyzed with flow cytometry. The MC3T3‐E1 or NIH3T3 cells were seeded at a density of 1×10^5^ cells per well on 24‐well plates, and were allowed to attach for 24 h. Then the sensors dispersed in 1 mL medium (125 µg mL^−1^) were added to each well. The cells and sensors were co‐incubated for 24 h in the incubator. Then media containing sensors were removed. After being washed with PBS three times to remove the free sensors, the cells were detached with trypsinization and dispersed in PBS at a concentration of about 1.6×10^5^ mL^−1^. Cells incubated with medium only under the same conditions were used as control samples. Triplicate samples were set for each group. The average fluorescence intensity (Ex 488 nm, Em 515–545 nm) per cell and the ratio of cells with fluorescence were measured with flow cytometry (FACS Calibur, BD). The average values of the triplicate samples in each group were analyzed.

### Statistical Analysis

In the cell viability assay, data were expressed as the mean ± SD. Statistical analysis was determined by one‐way analysis of variance (ANOVA) with the Origin software. Means comparison was performed with the Tukey's method. A *p* value < 0.05 was considered statistically significant.

## Conflict of Interest

The authors declare no conflict of interest.

## Supporting information

Supporting Information

Supplemental Video 1

Supplemental Video 2

Supplemental Video 3

Supplemental Video 4

Supplemental Video 5

## Data Availability

The data that support the findings of this study are available from the corresponding author upon reasonable request.
