# Supplementary material for: Submicron‐Sized In Situ Osmotic Pressure Sensors for In Vitro Applications in Biology
Source: Adv Healthc Mater. 2023 Jan 13;12(9):2202373. doi: 10.1002/adhm.202202373 (PMC11468766; doi:10.1002/adhm.202202373)
Supplement: Supplementary file 1 — Supporting Information [file ADHM-12-2202373-s002.pdf]

ADVANCED HEALTHCARE MATERIALS

Supporting Information

for *Adv. Healthcare Mater.*, DOI 10.1002/adhm.202202373

Submicron-Sized In Situ Osmotic Pressure Sensors for In Vitro Applications in Biology

Wenbo Zhang, Luca Bertinetti, Efe Cuma Yavuzsoy, Changyou Gao, Emanuel Schneck and Peter Fratzl**

Supporting Information

Submicron-Sized In Situ Osmotic Pressure Sensors for In Vitro Applications in Biology

Wenbo Zhang, Luca Bertinetti, Efe Cuma Yavuzsoy, Changyou Gao, Emanuel Schneck*, and Peter Fratzl*

W. Zhang, E. C. Yavuzsoy, P. Fratzl

Department of Biomaterials, Max Planck Institute of Colloids and Interfaces, 14476 Potsdam, Germany

E-mail: Peter.Fratzl@mpikg.mpg.de

E. Schneck

Department of Physics, Technische Universität Darmstadt, 64289 Darmstadt, Germany

and Department of Biomaterials, Max Planck Institute of Colloids and Interfaces, 14476 Potsdam, Germany

E-mail: emanuel.schneck@pkm.tu-darmstadt.de

L. Bertinetti

B CUBE Center for Molecular Bioengineering, Technische Universität Dresden, 01307 Dresden, Germany

and Department of Biomaterials, Max Planck Institute of Colloids and Interfaces, 14476 Potsdam, Germany

C. Gao

MOE Key Laboratory of Macromolecular Synthesis and Functionalization, Department of Polymer Science and Engineering, Zhejiang University, Hangzhou 310027, China

Supplementary results

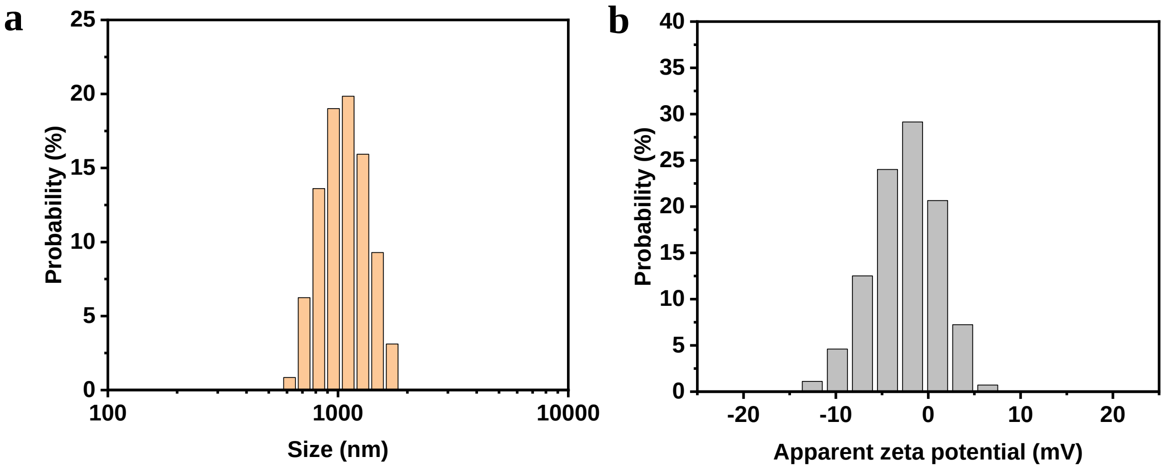

Figure S1. Distributions of size (a) and zeta potential (b) of Lip-DA-0.05 liposomes in water as obtained by dynamic light scattering (DLS) and phase analysis light scattering (PALS), respectively.

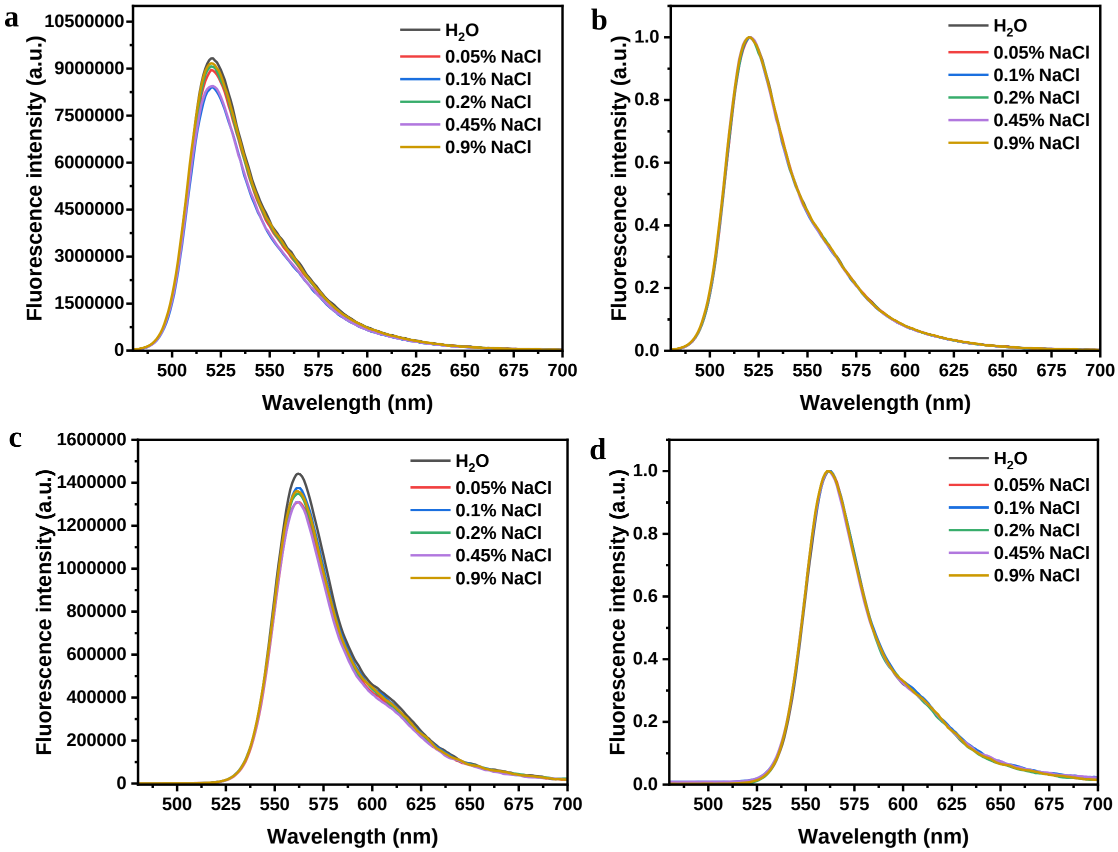

Figure S2. (a,c) Fluorescence emission spectra of ATTO 488 or ATTO 542 in water or 0.05%,0.1%,0.2%, 0.45% or 0.9% NaCl at a concentration of 1 µM. (b,d) Normalized fluorescence emission spectra in (a) and (c), respectively.

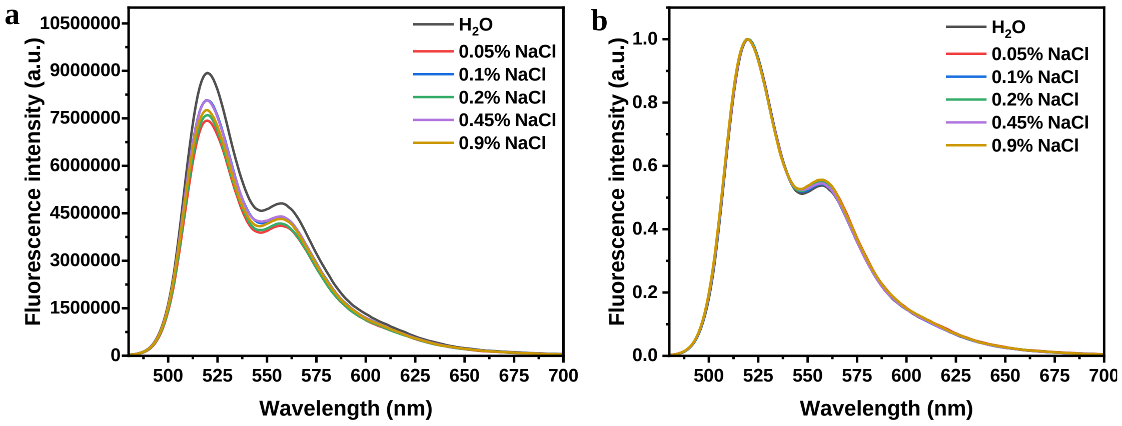

Figure S3. (a) Fluorescence emission spectra of ATTO 488-ATTO 542 mixture (1:1 molar ratio) in water or 0.05%,0.1%,0.2%, 0.45% or 0.9% NaCl at a concentration of 1 μM. (b) Normalized fluorescence emission spectra in (a).

Table S1. Diameter and zeta potential of Lip-PEG5 liposomes and initial FRET ratio R_0, slope, and sensing range for each liposome type in Figure S4.

Liposome type	Diameter [nm]	Zeta potential [mV]	Initial R_0 [%]	Slope [%/MPa]	Sensing range [MPa]
**Lip-PEG5-0**	364 ± 34	-38.3 ± 0.9			
**Lip-PEG5-DA-0**	332 ± 33	-37.5 ± 0.3	151.3	317.8	0 - 0.3
**Lip-PEG5-DA-0.05**	275 ± 25	-12.0 ± 0.9	75.9	63.7	0.05 - 0.95
**Lip-PEG5-DA-0.1**	263 ± 23	-9.5 ± 3.3	62.6	39.1	0.08-
**Lip-PEG5-DA-0.2**	309 ± 11	-7.8 ± 2.5	50.6	27.2	0.16-

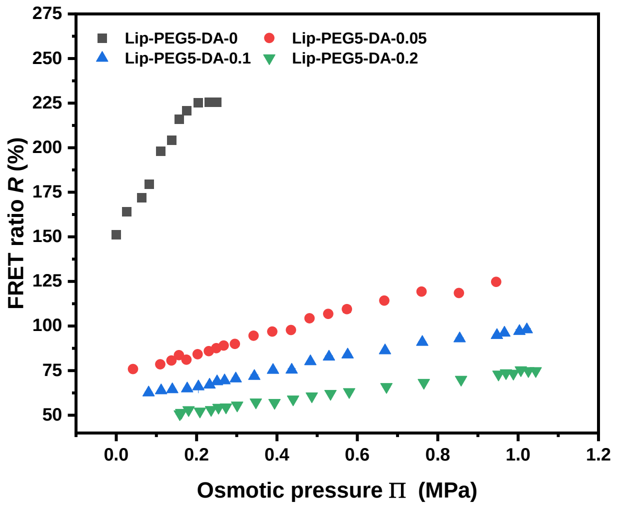

Figure S4. FRET ratio obtained with Lip-PEG5-DA liposomes loaded with H_2O, 0.05%, 0.1% and 0.2% NaCl and with a dye concentration of 50 µM (1:1 molar ratio) as a function of the external osmotic pressure generated by various concentrations of NaCl.

Table S2. Size and zeta potential of Lip-PEG10 liposomes.

Liposome type	Diameter (nm)	Zeta potential (mV)
**Lip-PEG10-DA-0**	217 ± 10	-42.1 ± 1.4
**Lip-PEG10-DA-0.05**	191 ± 10	-16.7 ± 0.4

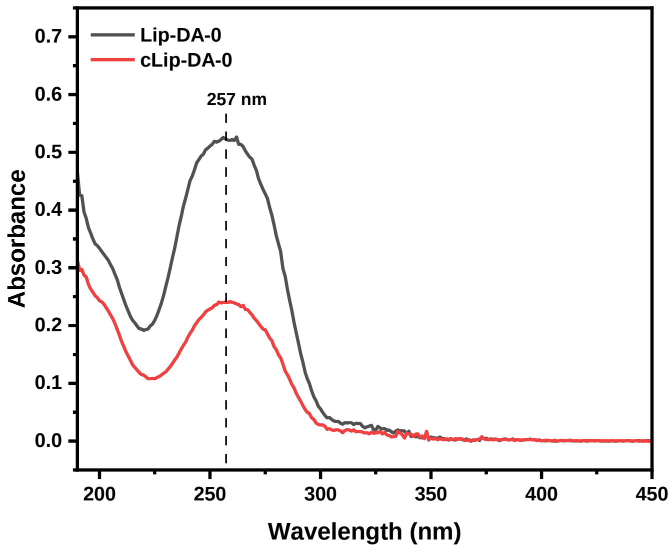

Figure S5. UV-Vis absorption spectra of Lip-DA-0 and cLip-DA-0 liposomes in water at a concentration of 0.01 mg mL^{-1}.

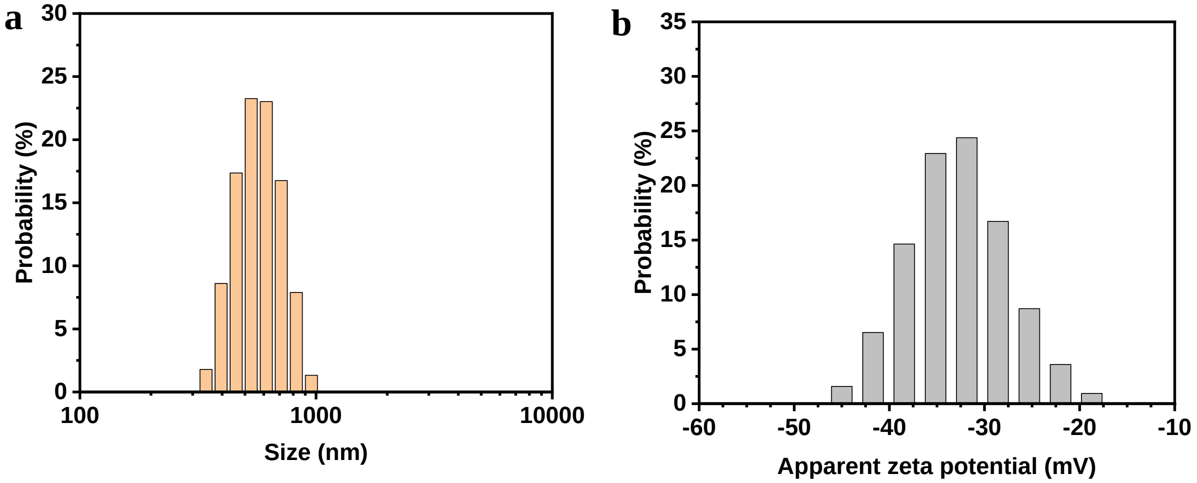

Figure S6. Distributions of size (a) and zeta potential (b) of cLip-DA-0 liposomes in water as obtained by DLS and PALS, respectively.

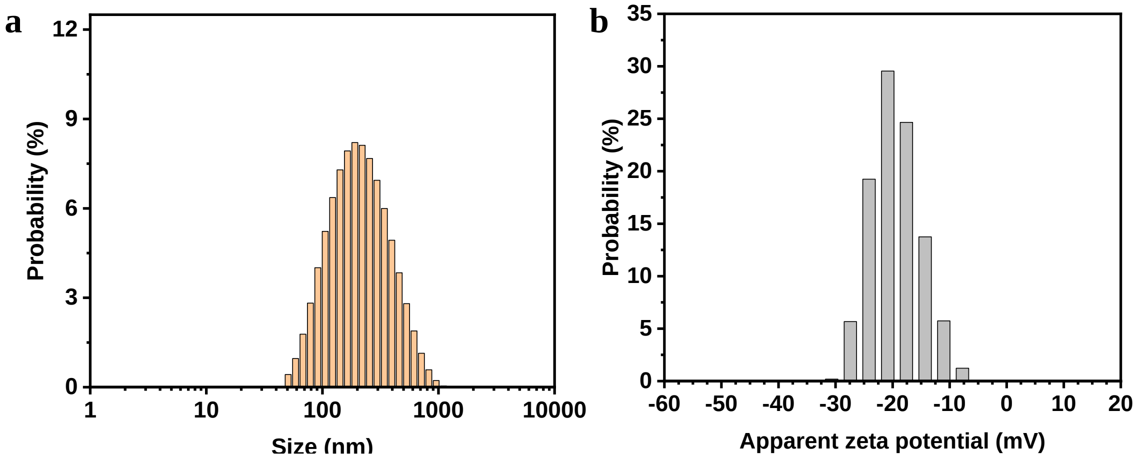

Figure S7. Distributions of size (a) and zeta potential (b) of cLip-PEG10-DA-0.05 liposomes in water as obtained by DLS and PALS, respectively.

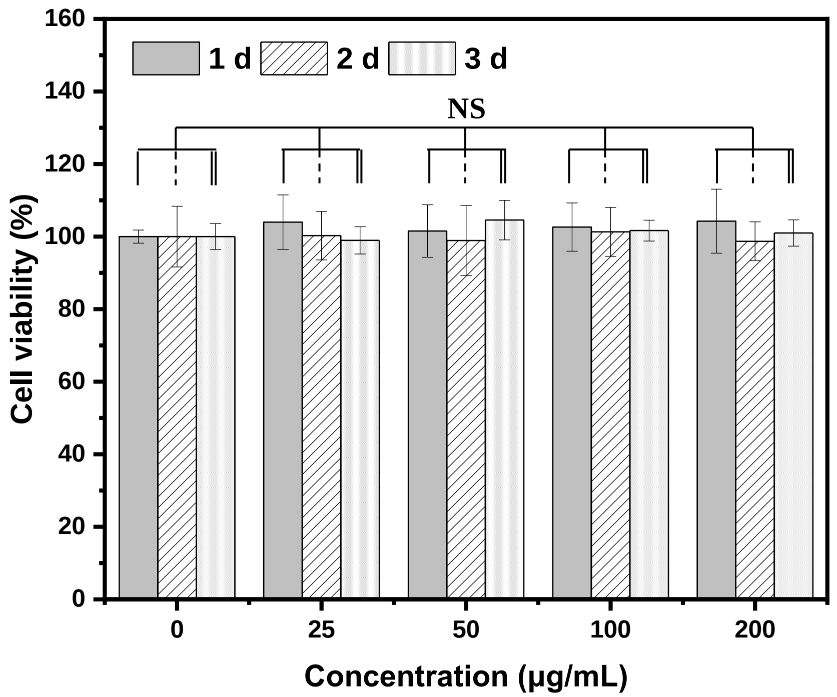

Figure S8. Viability of NIH3T3 cells after co-incubation with 25, 50, 100 and 200 μg mL^{-1} Lip-PEG10-DA-0.05 liposomes for 1, 2 and 3 d, respectively. Data are expressed as the mean ± standard deviation (SD), n = 5. NS indicates no significant difference at a level of $p < 0.05$.

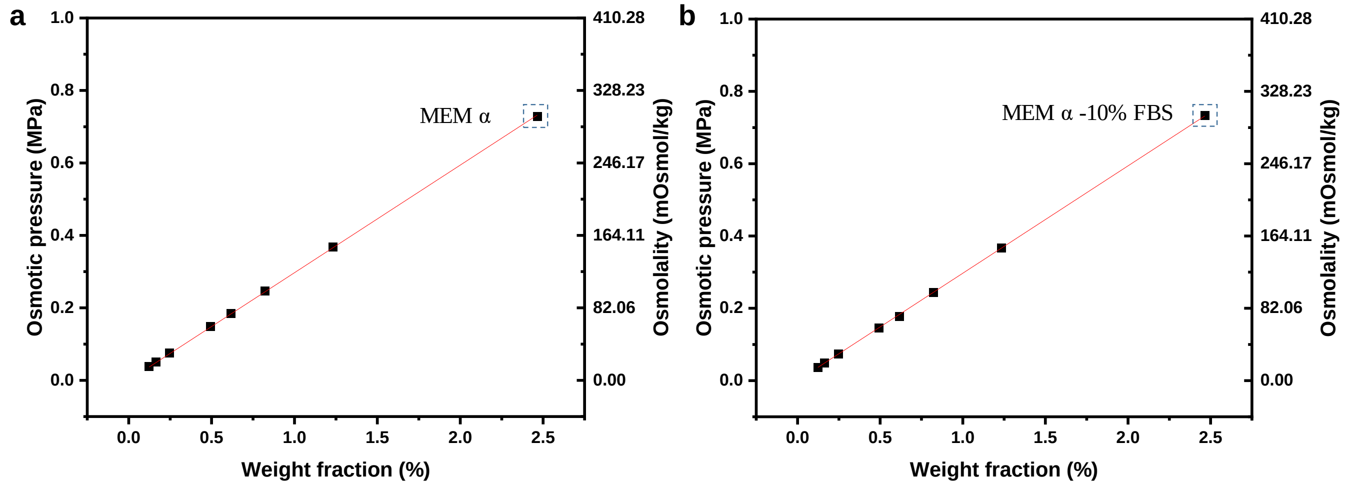

Figure S9. Osmolality and osmotic pressure variation of MEM α (or dilutions) (a) and MEM α-10%FBS (or dilutions) (b) measured with freezing point osmometer.

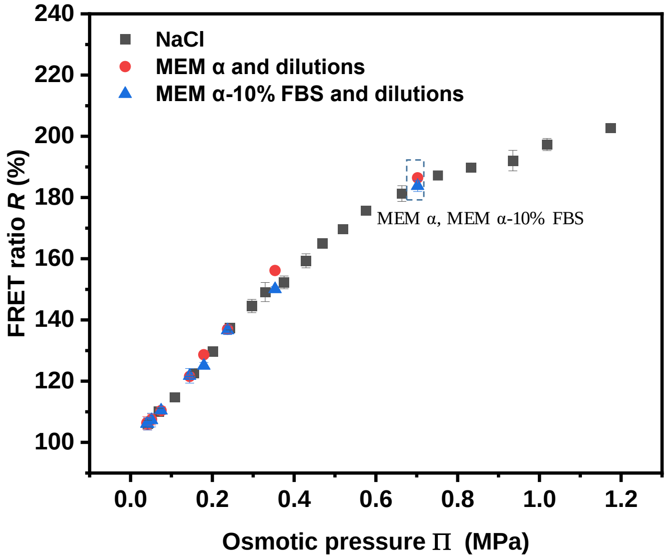

Figure S10. Osmotic responses of the sensors in cell culture media. FRET ratio obtained with Lip-PEG10-DA-0.05 liposomes with a dye concentration of 50 µM (1:1 molar ratio) as a function of the external osmotic pressure generated by various concentrations of NaCl, MEM α (or dilutions) and MEM α-10% FBS (or dilutions).

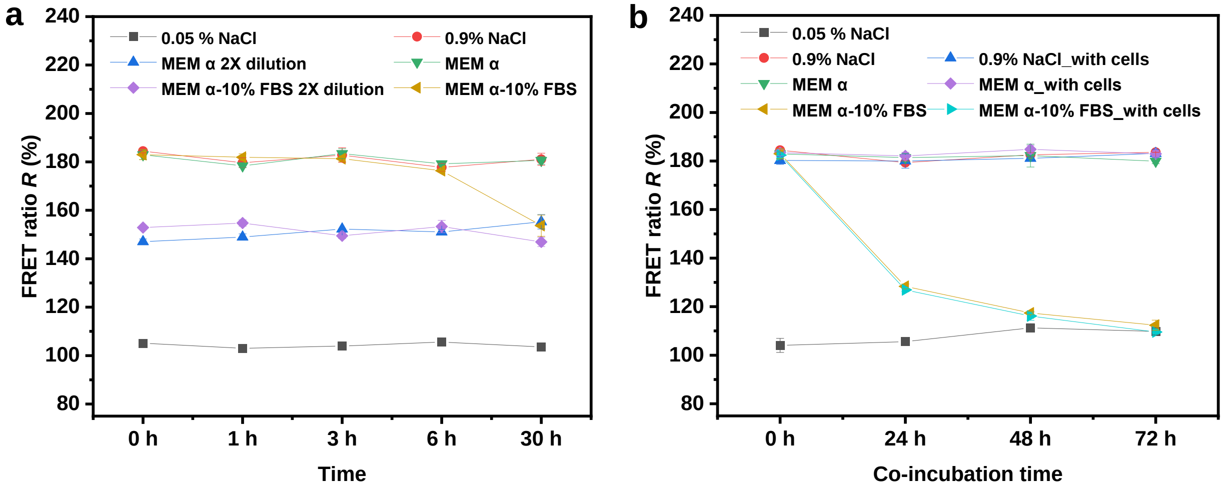

Figure S11. FRET ratio obtained with Lip-PEG10-DA-0.05 sensors with a dye concentration of 50 µM (1:1 molar ratio) after incubation in NaCl, MEM α and MEM α-10% FBS or in 2x diluted media: (a) without cells for 1, 3, 6 and 30 h at r.t. and (b) with/without MC3T3-E1 cells for 24, 48 and 72 h at 37 ^{o}C.

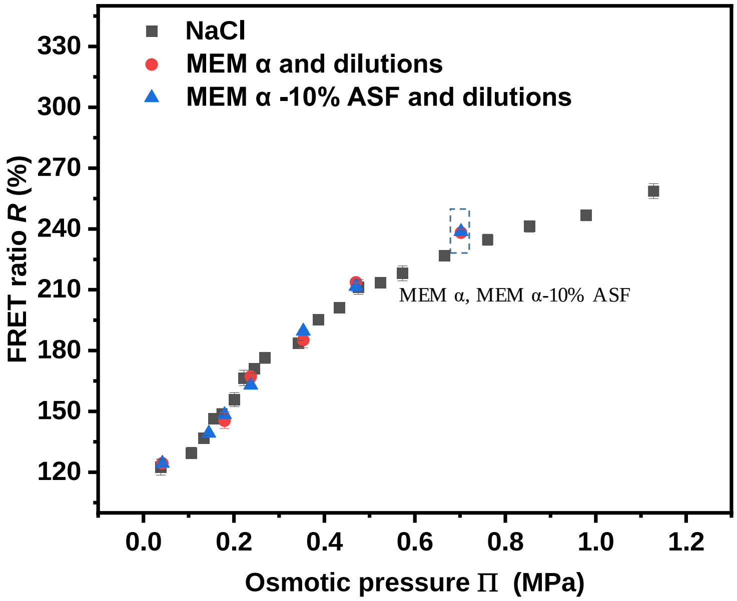

Figure S12. Osmotic responses of the sensors in cell culture media. FRET ratio obtained with Lip-PEG10-DA-0.05 liposomes with a dye concentration of 75 µM (1:1 molar ratio) as a function of the external osmotic pressure generated by various concentrations of NaCl, MEM α (or dilutions) and MEM α-10% ASF (or dilutions).

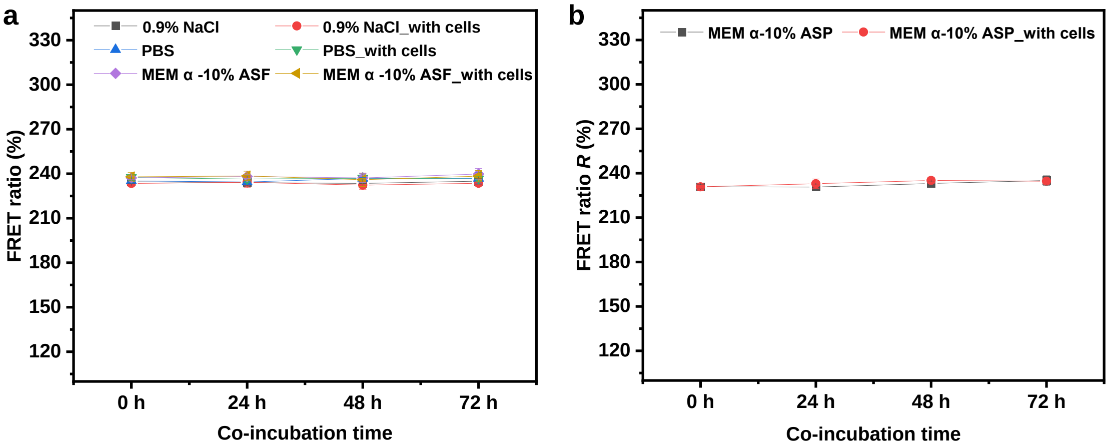

Figure S13. FRET ratio obtained with Lip-PEG10-DA-0.05 sensors with a dye concentration of 75 µM (1:1 molar ratio) after incubation in: (a) 0.9% NaCl, PBS or MEM α-10% ASF with/without MC3T3-E1 cells for 24, 48 and 72 h at 37 °C and (b) MEM α-10% ASP with/without MC3T3-E1 cells for 24, 48 and 72 h at 37 °C.

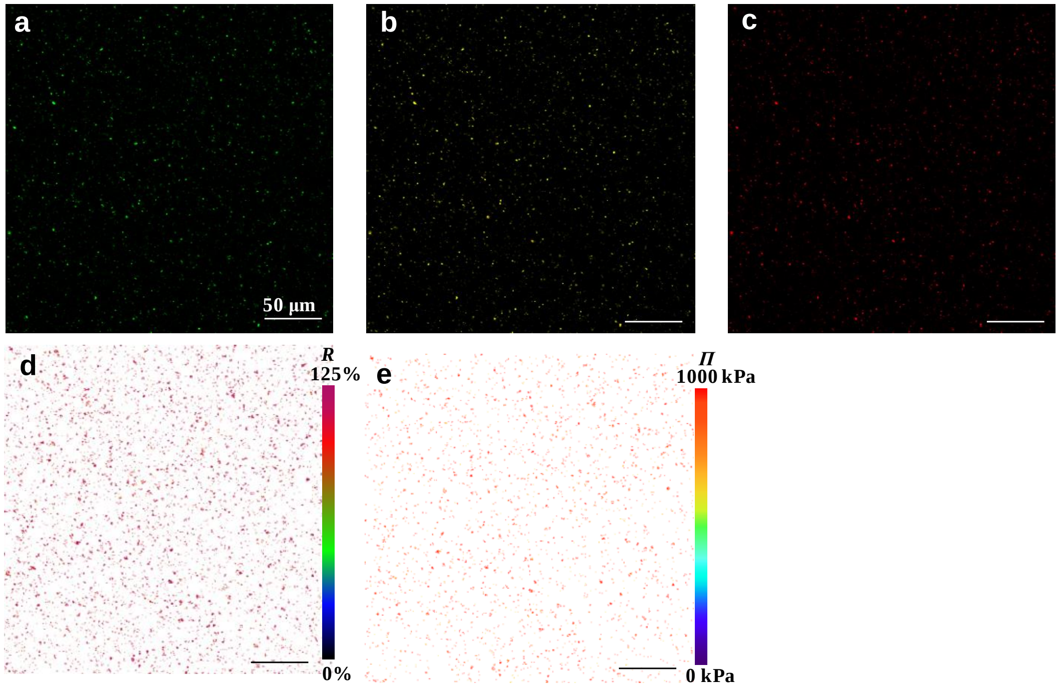

Figure S14. Application of Lip-PEG10-DA-0.05 sensors for osmotic pressure imaging in cell culture media. (a-c) Confocal laser scanning microscopy (CLSM) images of sensors (125 μg mL^{-1}) in MEM α-10% ASF without cells. The green (a), yellow (b) and red (c) fluorescence represent the donor emission signal (Ex 458 nm, Em 468–538 nm), the sensitized acceptor emission signal (Ex 458 nm, Em 571–700 nm) and the direct acceptor emission signal (Ex 561 nm, Em 571–700 nm), respectively. (d,e) The FRET ratio (d) and osmotic pressure (e) mapping with the sensors in MEM α-10% ASF without cells.

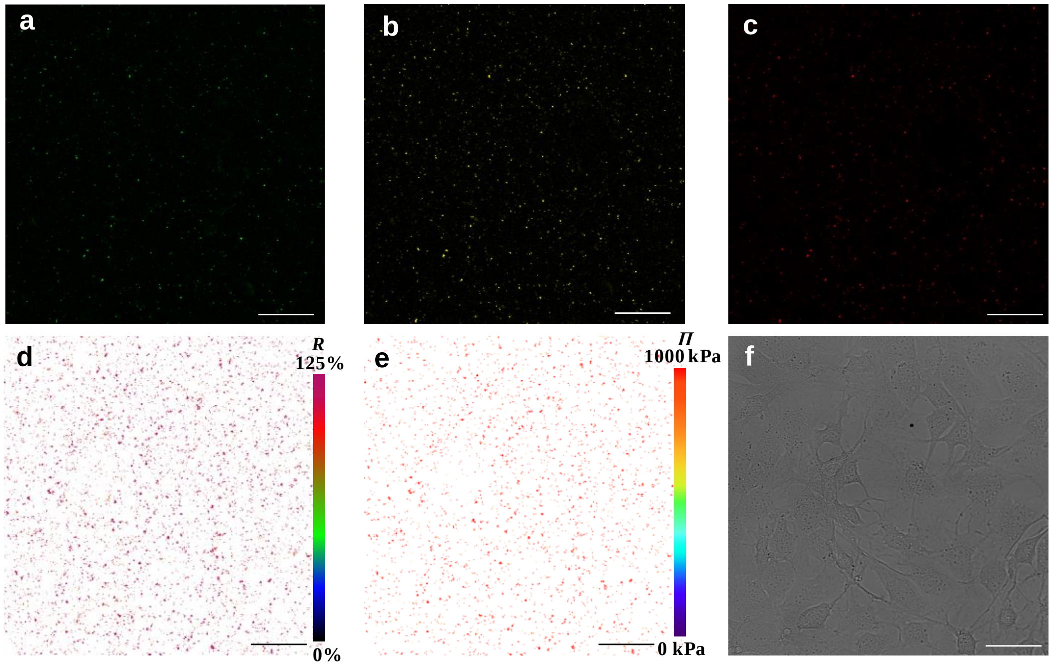

Figure S15. Application of Lip-PEG10-DA-0.05 sensors for osmotic pressure imaging in cell system. (a-c) CLSM images of sensors (125 μg mL^{-1}) in MEM α-10% ASF with MC3T3-E1 cells. The green (a), yellow (b) and red (c) fluorescence represent the donor emission signal (Ex 458 nm, Em 468–538 nm), the sensitized acceptor emission signal (Ex 458 nm, Em 571–700 nm) and the direct acceptor emission signal (Ex 561 nm, Em 571–700 nm), respectively. (d,e) The FRET ratio (d) and osmotic pressure (e) mapping with the sensors in MEM α-10% ASF with cells. (f) Bright field image of the MC3T3-E1 cells.

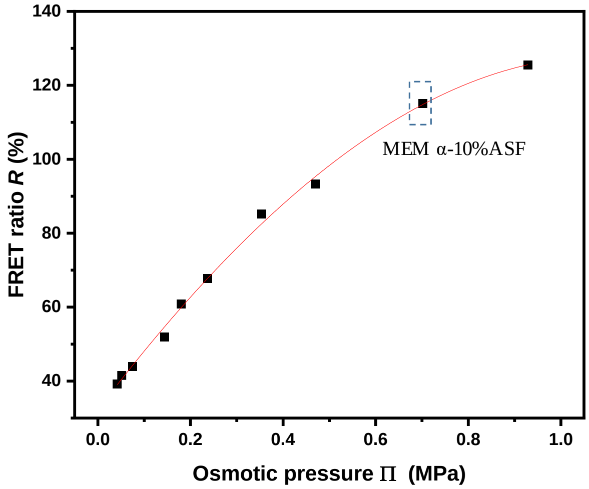

Figure S16. Application of Lip-PEG10-DA-0.05 sensors with a dye concentration of 75 μM (1:1 molar ratio) for osmotic pressure imaging. Calibration curve recorded at various osmotic pressures exerted by MEM α-10% ASF and the diluted media, or MEM α-10% ASF with added NaCl (the highest osmotic pressure was exerted by MEM α-10% ASF with additional 0.3% NaCl). The solid line is an empirical third-order polynomial fit to the data points (coefficient of determination = 0.997).

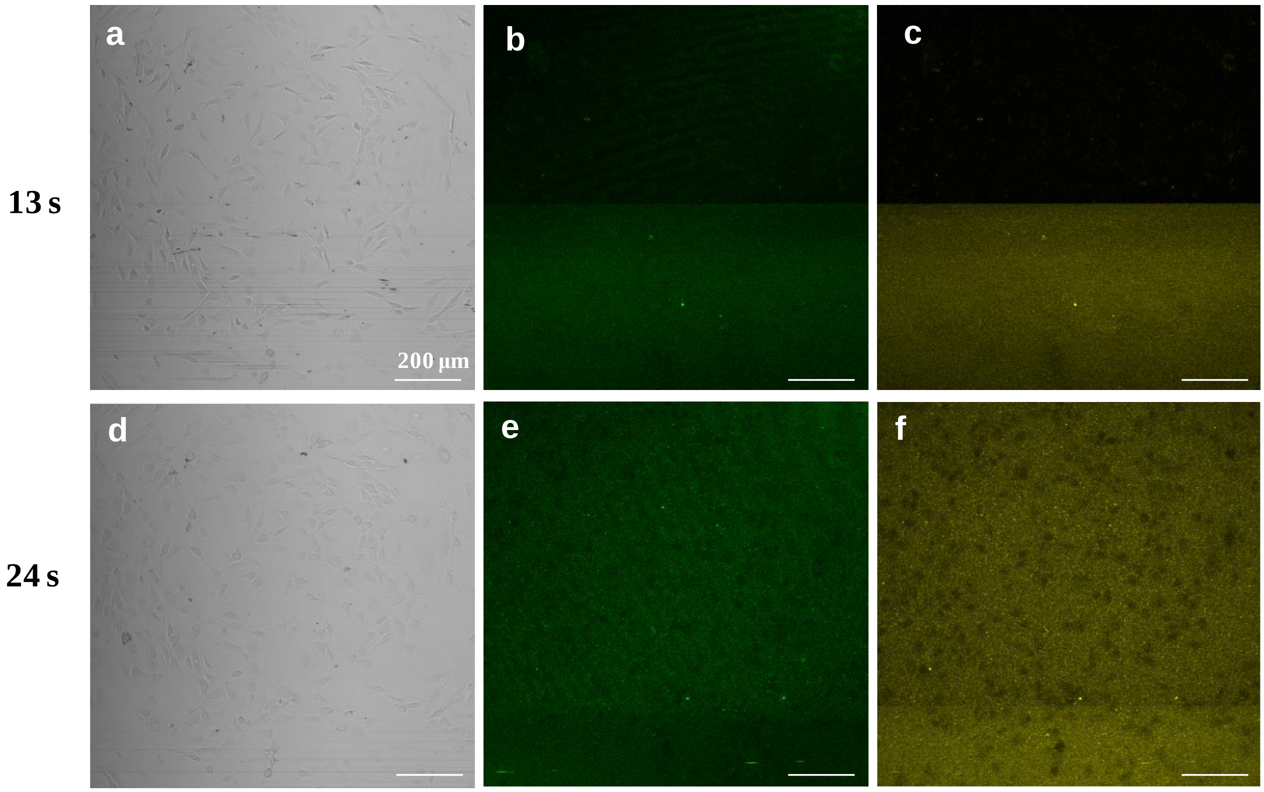

Figure S17. Application of Lip-PEG10-DA-0.05 sensors for in situ monitoring of osmotic pressure when changing cell culture media. (a-f) CLSM images show the moments when sensors/(MEM α-10% ASP medium) was added (a-c, 13 s in the Videos S1-S5) and when sensors/medium-0.3%NaCl was added (d-f, 24 s in the Videos S1-S5) to the MC3T3-E1cell culture. The concentration of the sensors was 125 µg mL^{-1}. (a,d) Bright field images of the live MC3T3-E1 cells. The green (b,e) and yellow (c,f) fluorescence represent the donor emission signal (Ex 458 nm, Em 468–538 nm) and the sensitized acceptor emission signal (Ex 458 nm, Em 571–700 nm), respectively.

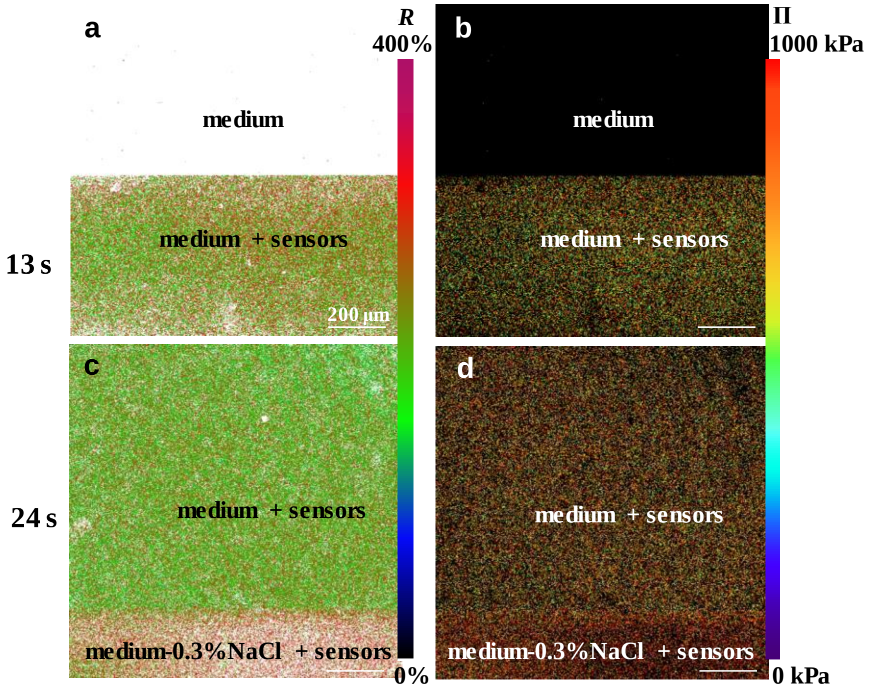

Figure S18. Application of Lip-PEG10-DA-0.05 sensors for in situ monitoring of osmotic pressure when changing cell culture media. (a-d) FRET ratio (a,c) and osmotic pressure (b,d) mapping images show the moments when sensors/(MEM α-10% ASP medium) was added (a and b, 13 s in the Videos S1-S5) and when sensors/medium-0.3%NaCl was added (c and d, 24 s in the Videos S1-S5) to the MC3T3-E1 cell culture. The concentration of the sensors was 125 μg mL^{-1}.

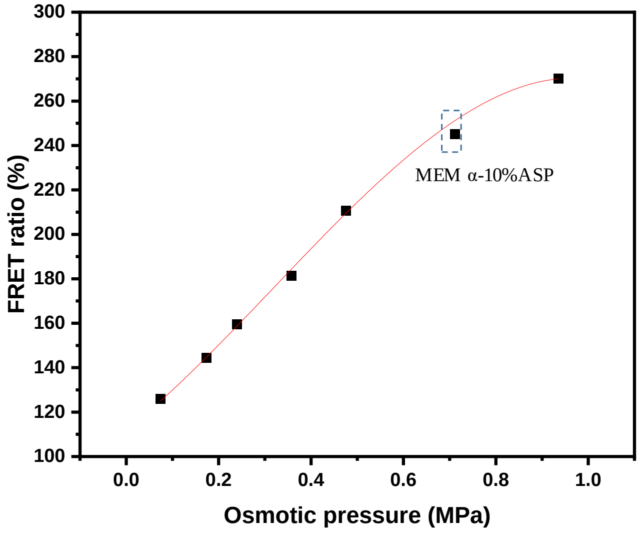

Figure S19. Application of Lip-PEG10-DA-0.05 sensors with a dye concentration of 100 μM (1:1 molar ratio) for in situ monitoring of osmotic pressure when changing cell culture media. Calibration curve recorded at various osmotic pressures exerted by MEM α-10% ASP and the diluted media, or MEM α-10% ASP with added NaCl (the highest osmotic pressure was exerted by MEM α-10% ASP with additional 0.3% NaCl). The solid line is an empirical third-order polynomial fit to the data points (coefficient of determination = 0.999).

Explanation of Videos S1-S5

Video S1, bright field images of the MC3T3-E1 cells; Video S2, donor channel images; Video S3, FRET channel images; Video S4, FRET ratio images; Video S5, osmotic pressure images.

Phases in the process of in situ monitoring of osmotic pressure when changing cell culture media: phase 1, 0-2 s, MEM α-10% ASP medium only; phase 2, 2-5 s, MEM α-10% ASP medium only is removed; phase 3, 5-10s, medium-0.3%NaCl only is added; phase 4, 10-13 s, medium-0.3%NaCl only is removed; phase 5, 13-21 s, sensors/(MEM α-10% ASP medium) is added; phase 6, 21-25 s, sensors/(MEM α-10% ASP medium) is removed; phase 7, 25-34 s, sensors/medium-0.3%NaCl is added.

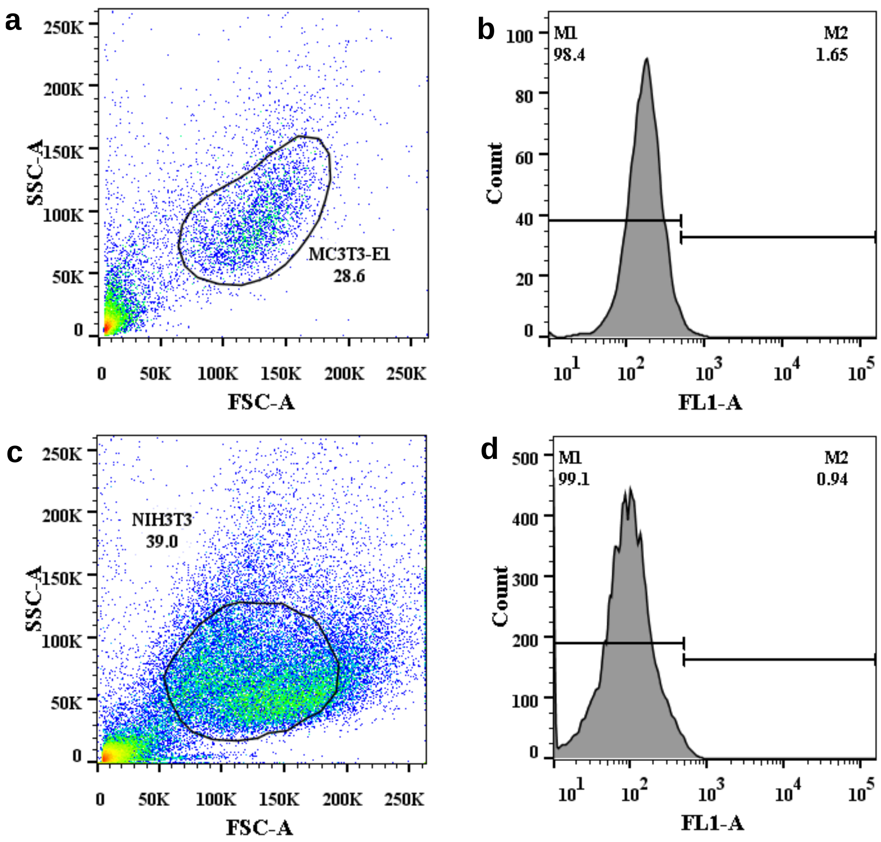

Figure S20. Flow cytometry plots showing the percentage of different cell subsets gated in MC3T3-E1 (a,b) or NIH3T3 (c,d) cells after co-incubation with 125 µg mL^{-1} sensors for 24 h at 37 °C: (a,c) cell size (forward scatter area, FSC-A) and granularity (side scatter area, SSC-A) were used to identify the main cell fraction; (b,d) analysis of cell fluorescence intensity (Ex 488 nm, Em 515–545 nm) in the main cell fraction in (a,c) revealed percentage of both the sensor-deficient (M1) and the sensor+ (M2) populations.

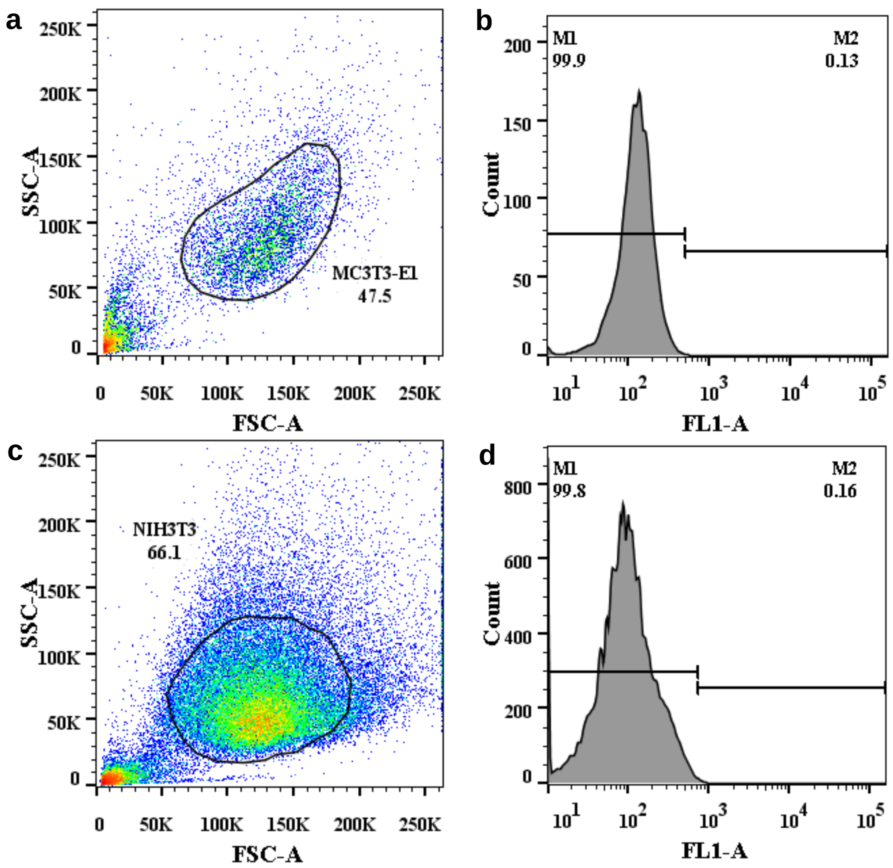

Figure S21. Flow cytometry plots showing the percentage of different cell subsets gated in MC3T3-E1 (a,b) or NIH3T3 (c,d) cells after culture without sensors for 24 h at 37 °C: (a,c) cell size (forward scatter area, FSC-A) and granularity (side scatter area, SSC-A) were used to identify the main cell fraction; (b,d) analysis of cell fluorescence intensity (Ex 488 nm, Em 515–545 nm) in the main cell fraction in (a,c) revealed percentage of both the sensor-deficient (M1) and the sensor+ (M2) populations.
